# Porcelain versus Porcelain Stoneware: So Close, So Different. Sintering Kinetics, Phase Evolution, and Vitrification Paths

**DOI:** 10.3390/ma16010171

**Published:** 2022-12-24

**Authors:** Sonia Conte, Chiara Molinari, Matteo Ardit, Giuseppe Cruciani, Michele Dondi, Chiara Zanelli

**Affiliations:** 1 CNR-ISSMC, Institute of Science, Technology and Sustainability for Ceramics, 48018 Faenza, Italy; 2 Department Physics and Earth Sciences, University of Ferrara, 44122 Ferrara, Italy

**Keywords:** porcelain, porcelain stoneware, sintering, phase composition, mullite, microstructure, non-crystalline matrix

## Abstract

Five porcelain and porcelain stoneware bodies were investigated to compare sintering mechanisms and kinetics, phase and microstructure evolution, and high temperature stability. All batches were designed with the same raw materials and processing conditions, and characterized by optical dilatometry, XRF, XRPD-Rietveld, FEG-SEM and technological properties. Porcelain and porcelain stoneware behave distinctly during sintering, with the convolution of completely different phase evolution and melt composition/structure. The firing behavior of porcelain is essentially controlled by microstructural features. Changes in mullitization create conditions for a relatively fast densification rate at lower temperature (depolymerized melt, lower solid load) then to contrast deformations at high temperature (enhanced effective viscosity by increasing solid load, mullite aspect ratio, and melt polymerization). In porcelain stoneware, the sintering behavior is basically governed by physical and chemical properties of the melt, which depend on the stability of quartz and mullite at high temperature. A buffering effect ensures adequate effective viscosity to counteract deformation, either by preserving a sufficient skeleton or by increasing melt viscosity if quartz is melted. When a large amount of soda–lime glass is used, no buffering effect occurs with melting of feldspars, as both solid load and melt viscosity decrease. In this batch, the persistence of a feldspathic skeleton plays a key role to control pyroplasticity.

## 1. Introduction

The terms *porcelain* and *porcelain stoneware* are referring to dense and largely vitrified ceramic materials, which are utilized in distinct applications. Porcelain is mainly addressed, with its several variants, to tableware, sanitaryware, electric insulators, and artware [1,2]. Porcelain stoneware (sometimes *porcelainized stoneware*) is essentially employed for wall and floor tiles, large slabs, and laboratory and kitchen tops [3]. Formulations are always poor in iron oxide, resulting in light-colored bodies, sometimes grouped together under the term *whiteware* [4].

Both materials are prepared from different proportions of the same ingredients (i.e., kaolin, ball clay, feldspar, quartz). Porcelain batches are made of raw materials with well-established technological roles [1]: plastic component (kaolin, ball clay); flux (potassic and mixed K-Na feldspars, nepheline syenite); and filler (quartz, chamotte, alumina). For this reason, porcelain bodies are often referred to as *triaxial batches* [2]. Porcelain stoneware bodies are more flexible in designing, and are usually formulated on the dichotomy between plastic (ball clay, sometimes kaolin or pyrophyllite) and non-plastic (mostly fluxes, i.e., sodic and mixed Na-K feldspars, quartz–feldspathic sands, etc.) raw materials [3]. Filler is added as a minor ingredient since quartz is commonly provided to some extent by fluxes and clay materials [4].

Differences in batch designing reflect distinct technological requirements imposed by ceramic processing, especially in the shaping and firing stages. Porcelain bodies are generally processed by slip casting, roller-head machine, or isostatic pressing [1], while porcelain stoneware tiles are manufactured by uniaxial pressing, roll compaction, or extrusion [5]. Porcelain requires firing schedules lasting several hours, typically a single-firing in the 1250–1300 °C range for sanitaryware, or a double-firing in the case of tableware: biscuit at 900–1000 °C, and gloss at 1250–1400 °C, depending on the body type (hard or soft porcelain) [1,2,3,5]. At variance, porcelain stoneware tiles are produced by fast single-firing at 1190–1230 °C as maximum temperature, with schedules lasting around 1 h cold-to-cold, extending to 2–3 h in case of very thick large slabs [5].

Despite the aforementioned differences, the terms *porcelain stoneware* and *porcelain* are sometimes (mistakenly) interchanged. Some companies have begun shortening ‘porcelain stoneware tiles’ into ‘porcelain tiles’ for marketing reasons, and this practice has spread in the scientific literature, causing misunderstandings. Indeed, a formal distinction between porcelain and porcelain stoneware ceramics is lacking in the scientific literature, so confusion is fueled by the absence of standard criteria to clearly discriminate the two materials. This circumstance may induce the questionable impression that porcelain stoneware and porcelain are just variations of the same material with gradual changes in technological behavior, microstructure, and phase composition.

The present study was undertaken to clarify the latter point, and to gain an in-depth understanding of the reasons behind the different firing behavior of whiteware batches, shedding light on the microstructural mechanisms governing sintering kinetics and vitrification path of porcelain stoneware compared with porcelain.

## 2. Materials and Methods

Five batches were designed to reproduce typical industrial bodies for tableware, sanitaryware, and floor tiles. 

In particular, two porcelain-like materials were prepared (a soft porcelain, SOPO, and a vitreous china, VICH) together with three kinds of porcelain stoneware: two classic bodies (POST and GPOR) and a “glass-ceramic stoneware” (GSTO), the latter taking into account new ceramic batches obtained by recycling glass cullet in the context of the circular economy [6,7,8,9,10,11,12,13,14,15,16,17,18,19,20,21]. POST and GPOR batches were formulated to appraise the effects of a different ball-clay-to-kaolin ratio, and a different source of sodic feldspar. In order to constrain the well-known effects of the Na/K ratio [22,23] and silica amount [24] on the technological behavior and phase composition, all batches were based on the same raw materials: ball clay, kaolin, sodic feldspar, and quartz sand (Table 1). An alternative sodic feldspar (B) was used in the GPOR formulation. In addition, a soda–lime glass, currently utilized in tile-making, was employed in the GSTO formulation. The chemical composition of the five raw batches was determined by X-ray fluorescence (EDXRF S2 PUMA, Bruker). All batch formulations and compositions are reported in Table 2. It is worth noting that using the same raw materials for all batches has hindered the replication of the industrial compositions for the porcelain samples, which are usually formulated with a K_2_O/Na_2_O ratio higher than that of SOPO. For this reason, the behavior of potassic bodies will be investigated in a future work. 

The five batches were experimented at the laboratory scale, simulating the industrial tile-making process. The raw materials were mixed by wet milling in a porcelain jar using dense alumina media for 15 min, with a resulting mean particle diameter between 2.7 and 3.5 µm (Table 2). The slips were oven-dried at 105 ± 5 °C, de-agglomerated (hammer mill with grid of 500 µm) and manually granulated (sieve 2 mm, powder moisture ∼7–8 wt%). Powders were pressed (40 MPa) into 50 mm diameter discs, dried in an electric oven at 105 ± 5 °C overnight and characterized for: particle size distribution by X-ray monitoring of gravity sedimentation (ASTM C958) (Figure S1), specific weight of powders by helium pycnometry (ASTM C329), bulk density (weight/volume ratio) and relative density, calculated as bulk density/specific weight ratio (Table 2).

Discs were fired in an electric kiln at maximum temperatures from 1000 to 1250 °C with a thermal cycle of about 1 h cold-to-cold. Further firings up to 1330 °C were carried out for VICH and SOPO, with a slow thermal cycle of about 24 h cold-to-cold. In order to make comparable the results of firings with different heating rates and duration, Bullers rings (Ferro, series 75) were used. The difference in temperature, so estimated, is approximately 80 °C higher in the slow schedule with respect to the fast one. The fired products were characterized to determine the following technological properties: water absorption, open porosity (OP) and bulk density (BD) (ISO 10545-3); total porosity by TP = (1-BD/SW) × 100; closed porosity as CP = TP-OP; linear firing shrinkage, as (*D_m_-D_f_*)/*D_m_* × 100, where *D_m_* and *D_f_* are the diameter of the mould and the fired tile, and specific weight of powders (SW) by helium pycnometry (ASTM C329). All these results are reported in Table S1. The temperature necessary to reach a water absorption <0.5% (prescribed by the standards ISO 13006 and ISO/DIS 5644) was taken as the optimal firing (or *gresification*) temperature of each batch.

Quantitative phase analysis (QPA) was performed on selected fired samples by X-ray powder diffraction (Bruker D8 Advance, Karlsruhe, Germany, Bragg-Brentano geometry with a Cu X-ray tube operating at 40 kV and 40 mA). XRPD data were collected by a silicon strip detector (LynxEye, Bruker) from 10–100 °2θ, with a step size of 0.02 °2θ, and a counting time of 1 s per step (collected by means of a Si (Li) solid-state detector set to discriminate Cu Kα1,2 radiation). Prior to XRPD measurements, each sample was admixed with 20 wt% corundum as internal standard (traced to NIST 676a) for the quantification of the crystalline phases and XRD amorphous content [25]. The identification of the crystalline phases (qualitative phase analysis) was performed with the Diffrac.Suite EVA software. Subsequently, XRPD patterns were modelled by a full profile Rietveld refinement with the GSAS-EXPGUI software package [26,27]. Up to 40 independent variables were refined: phase fractions, zero point, 25–30 coefficients of the shifted Chebyschev function to fit the background, unit cell parameters, profile coefficients (one Gaussian, G_w_, and one Lorentzian term, L_x_). The starting structural models employed during the Rietveld refinement were downloaded from the Inorganic Crystal Structure Database (ICSD), the world’s largest database of completely determined inorganic crystal structures. The agreement indices, as defined in GSAS, for the final least-squares cycles of all refinements are represented by Rp (%), Rwp (%), Χ2 and R(F2) (%). For the refined patterns, they were found in the following ranges: 9.0% < Rp < 13.0%; 13.0% < Rwp < 15.0%; 2.5 < Χ2 < 4.5; and 10.0% < R(F2) < 13.0% (Figure S2). The chemical composition of the vitreous phase was calculated by the difference between the bulk chemical composition of the fired body and that derived from the contribution of crystalline phases, assuming their stoichiometric compositions weighted on the QPA [23]. Such a vitreous phase (deriving from the partial melting of the mineralogical phases constituting the ceramic bodies during the sintering process), contains elements that differently affect both structure and properties of the glass network. In order to facilitate data interpretation, some parameters were used to express specific structural features of the melt:

- degree of melt depolymerization (NBO/T, mol%) defined as the number of non-bridging oxygens (NBO) per tetrahedrally-coordinated cations (Si, Al), calculated from the composition of the vitreous phase [28];
- alumina saturation index of the melt, ASI [mol%] = Al_2_O_3_/(Na_2_O + K_2_O + CaO), representing the Al_2_O_3_ content not provided by feldspars [23];
- charge compensators (CCAT), alkali and alkaline-earths that compensate the Si^4+^ –Al^3+^ charge mismatch and stabilize Al ions in four-fold oxygen coordination. CCAT [mol%] = Na + K + 2Ca + 2Mg (up to a maximum value = Al) [29];
- glass network formers (GNF) which confer the polymeric structure to the melt, calculated from the composition of the vitreous phase as GNF [mol%] = Si + CCAT, corresponding to Al^3+^ charge compensated by alkali and alkaline earths [23,29];
- glass network modifiers (GNM) which cause the breakage of bonds between GNF and O, leading to a depolymerization of the melt network, composed either by alkali and alkaline earths or by Al^3+^; calculated from the composition of the vitreous phase as alkali and alkaline earths exceeding CCAT, i.e., GNM [mol%] = Na + K + 2Mg + 2Ca − (corresponding values in CCAT); or calculated as Al^3+^ in excess of CCAT, when it assumes an oxygen coordination higher than four or gives rise to Si–Al *triclusters* [28,30].

In addition, the speciation of aluminium was calculated from the phase composition of fired bodies (amount of Al in mullite and feldspars) and the chemical composition of vitreous phase (Al as GNF and GNM) [29,31].

The physical properties of the non-crystalline phase at high temperature were estimated by predictive models based on its chemical composition. The shear viscosity was calculated based on the Giordano and co-workers’ model [32], and the gas–liquid surface tension was estimated interpolating the data obtained by Appen’s [33] and Dietzel’s [34] methods.

Moreover, the effective viscosity of the body (η_eff_) was calculated as the product of the shear viscosity of the melt (η_melt_) by the relative viscosity (η_rel_), i.e., η_eff_ = η_rel_ ⋅ η_melt_ [23,29]. The relative viscosity was estimated by the relation: η_rel_ = (1 − Φ)^−m^, where Φ is the solid/crystalline fraction and *m* is the shape factor as proposed by Boccaccini [35]. This factor takes into account the effect of the shape of the crystalline particles by using the aspect ratio (AR) of each phase estimated by microstructural observations. A weighted average AR of the solid load was calculated from the AR and the weight fraction of each crystalline phase. The assignment of phases to the crystals visible in SEM images was performed by EDS analyses, point analyses as well as elemental distribution maps (not reported here).

The microstructure of the ceramic batches fired at the gresification temperature was observed by a scanning electron microscope (FEG-SEM, Zeiss, Sigma, gold sputtered polished surface), and images were collected using backscattered electrons. For the analysis of phase assemblages and morphology, the polished surfaces were etched for 4 min with a 15% HF solution, washed ultrasonically with distilled water, dried and subsequently coated with Au. Secondary electron images (SEI) were collected for the microstructure examination [5].

The sintering behavior was also evaluated by hot stage microscopy, using an optical thermo-dilatometer (TA, ODP868, Germany) which registered the size variation of a 5 × 5 × 5 mm chip cut from the dry samples, determined by the pixel count during a thermal cycle. The tests were run under isothermal conditions at different maximum temperatures between 1000 °C and 1250 °C, with a gradient of 80 °C × min^−1^ and dwell time of 30 min. Results were expressed in terms of shrinkage (area %) and relative density as a function of time [23,36]. The relative density was calculated by the starting density (bulk density/specific weight of powders) and the volumetric shrinkage during firing. Different stages of the sintering process were identified with reference to Figure S3. Isothermal sintering rates were calculated from early, linear and decreasing shrinkage (derived from specimen height variation) by dwell time at the various maximum temperatures. These *in-situ* tests also allowed the determination of other fundamental sintering parameters, such as densification extent and rate in the different stage of the process, the efficiency of densification and the de-sintering stage, as described in Figure S3 and reported in Table S2.

## 3. Results and Discussion

### 3.1. Sintering Mechanisms and Kinetics

**Figure 1:**
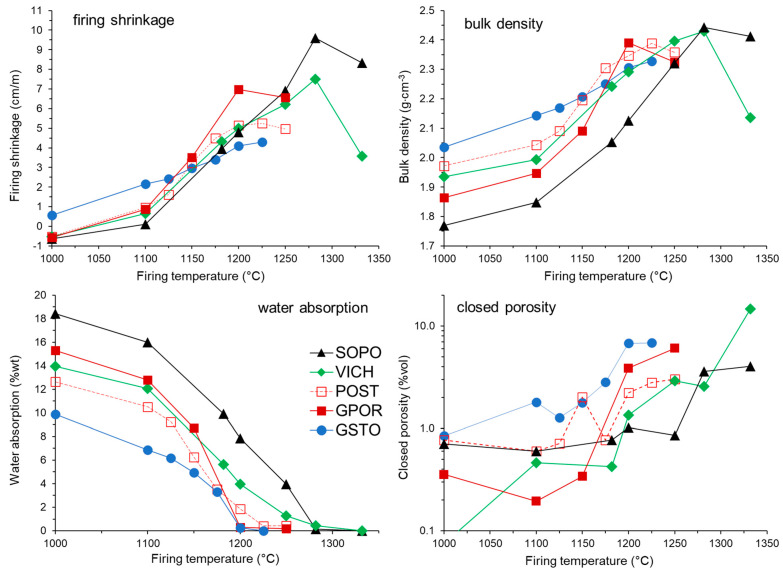
Firing behaviour of the ceramic bodies (*ex-situ* experiments).

The firing behavior of the ceramic bodies can be summarized through the evolution of their technological properties over a range of firing temperatures, as reported in Figure 1 (and Table S1). The five bodies show similar trends but with some differences, mostly related to the starting technological properties and the temperature at which the sintering process is accomplished. As far as the firing shrinkage is concerned, at 1000 °C, all batches have values lower than zero, indicating the intrinsic thermal expansion of the bodies prior to the start of the densification process. From 1100 °C onwards, the slopes of the curves become very steep, mirroring a strong increasing in the shrinkage degree with temperature. Once the maximum shrinkage is reached (with different values for each body, larger for porcelain than porcelain stoneware), an inversion of the curves due to the expansion of the bodies is observed, especially for porcelain. The above-described shrinkage features are valid for all batches but the glass–ceramic stoneware. This body is characterized by a certain degree of shrinkage already at 1000 °C, and a shrinkage variation that scales linearly with temperature.

The bulk density at 1000 °C substantially reflects what each ceramic body has inherited from the unfired compacts (Table 2), in the order: GSTO > POST > VICH > GPOR > SOPO, mirroring the compressibility of the starting batches. Also in this case, there is an increase in the sintering kinetics (apart from GSTO) from 1100 °C up to the maximum densification, which occurs at different temperatures: 1200 °C for GPOR, 1225 °C for POST and GSTO, 1280 °C for VICH and SOPO. Beyond these temperatures there is a bulk density decrease, most accentuated for VICH, attributable to the so-called “bloating” effect (i.e., pore volume expansion). As expected, porcelain bodies reach a maximum bulk density slightly higher than that of porcelain stoneware and glass-bearing stoneware [37]; it ranges from 2.328 g⋅cm^−3^ for GSTO to 2.443 g⋅cm^−3^ for SOPO (Table S1). The trend of water absorption is inversely related to bulk density. In all bodies, water absorption decreases with the sintering temperature, reaching the value prescribed for vitrified products (<0.5 wt%) in correspondence with the temperature of maximum densification. The only exception is represented again by GSTO, which reaches a satisfactory water absorption at 1200 °C, i.e., 25 °C lower than the maximum densification. Similarly, the closed porosity follows the evolution of the densification process, but with a positive correlation, increasing with the bulk density and temperature. GSTO is characterized by the highest values of closed porosity and lower bulk density of the set at the optimal firing temperature. In addition, VICH shows a significant increase of the closed porosity between 1280 and 1330 °C, matching the decrease in bulk density, as a consequence of de-sintering process and related bloating phenomena. Generally speaking, the technological properties for both porcelain and porcelain stoneware follow a similar evolution. The main difference, as known [1,3,23,37], stems from the temperature of optimal firing, which is higher for porcelain (1280 °C for VICH and SOPO) than for porcelain stoneware (1200 °C for GSTO and GPOR, and 1225 °C for POST). This difference mainly derives from the batch formulation (Table 2), being porcelain composed of a higher content of refractory raw materials (i.e., kaolin) and a lower proportion of fluxes (i.e., feldspars). The peculiar behavior of the glass–ceramic stoneware (higher shrinkage and bulk density corresponding to a lower water absorption in the 1000–1125 °C range), indicates an early activation of the densification process with respect to the other bodies. Anyway, it is the only batch with a mismatch between the temperature of maximum densification and minimum water absorption. Moreover, it is characterized by the lowest maximum bulk density of the set, coupled with the highest closed porosity. All these features are typical of porcelain stoneware bodies containing SLS glass in amount >10 wt% [6,11,20,38].

**Figure 2:**
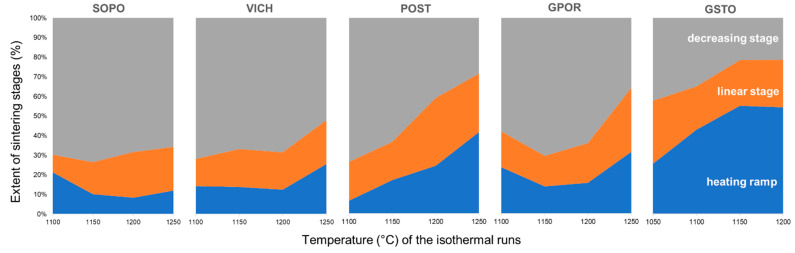
Extent of densification stages duration vs. temperature (°C) of isothermal runs.

Ceramic bodies sintering mechanisms were also investigated by *in-situ* experiments through hot stage microscopy under isothermal conditions. The main parameters are reported in Table S2. The temperatures at which the densification starts are quite close for SOPO and VICH, with an average value of 1045 ± 10 °C, in all the isothermal runs (1000–1150–1200–1250 °C), whereas porcelain stoneware bodies show some differences. In detail, POST is characterized by temperatures similar to that of porcelain bodies (1054 ± 22 °C), while GPOR and particularly GSTO exhibit the lowest temperatures of viscous flow activation (1006 ± 22 °C and 957 ± 17 °C, respectively). With reference to the main stages of densification (i.e., early, linear, and decreasing, as highlighted in Figure S3), the behavior of the five bodies also differs in terms of the sintering stage extent (Figure 2). 

During the heating ramp (in non-isothermal conditions), the densification of the two porcelain SOPO and VICH represents a small fraction over the total densification (8–26%), while the extent of this stage grows for porcelain stoneware, and it exceeds 50% for the glassy waste-bearing batch at temperatures >1150 °C. When compared at the same temperature, the extent of the linear stage is similar for every batch (16–23% of total densification at 1150 °C, 19–24% at 1200 °C, and 22–33% at 1250 °C), except for POST at 1200 °C, where this stage accounts for 35% of the total process. The extent of the decreasing stage is predominant for porcelain (52–73%), while for porcelain stoneware there is a growing impact of the densification occurring during the heating ramp with the increasing temperature, making shorter the final densification step, in the order GPOR < POST < GSTO.

**Figure 3:**
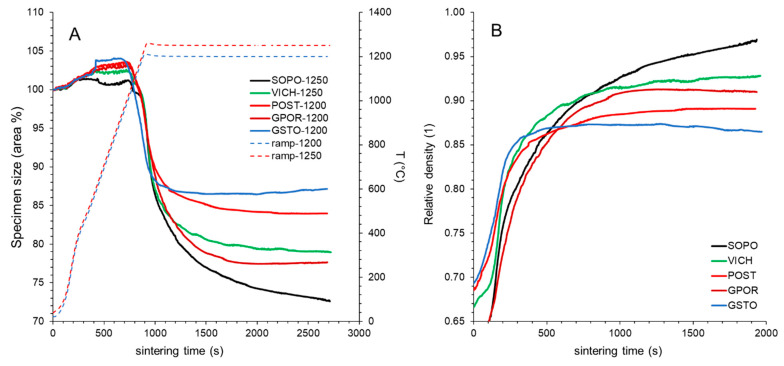
Firing behavior by HSM data (*in-situ* experiments): (**A**) specimen size vs. total sintering time; (**B**) relative density vs. time from the densification start.

The temperature at which the densification begins and the extent of the different sintering stages influence the efficiency of densification (expressed as maximum relative density), as well as the start of de-sintering process. The main features of this process can be appraised by inspecting the isothermal curves at temperatures as close as possible to those of optimal firing for each batch (i.e., 1200 °C for POST, GPOR and GSTO; 1250 °C for SOPO and VICH) (Figure 3). 

The densification proceeds initially with similar behavior for all batches, but important differences emerge in the final part of the curves in relation to:

- the switch point, representing the point at which the curve changes its inclination, passing from a linear to a decreasing densification stage;
- the degree of densification (i.e., the maximum size variation);
- the dimensional stability when dwell time increases.

Based on these data, curves in Figure 3A,B can be divided into two groups: (I) POST and GSTO with a delayed switch point (occurring at high relative density of 0.81-0.83, Table S2) and a low degree of densification, associated to a low dimensional stability with a certain bloating (5.2–6.4%, Table S2); (II) SOPO, VICH and GPOR, where the switch point is anticipated (at a lower relative density 0.73–0.79) and most of the densification process occurs in the decreasing stage (52–65%, Figure 2), resulting in a higher degree of densification and also stability. Porcelain batches, in fact, do not show any de-sintering phenomena at dwell time of 30 min, while GPOR is characterized by a low bloating degree (3.3%). The behavior observed for POST and GSTO was already recorded for porcelain stoneware containing waste glasses [29]. The presence of soda–lime glass allowed lowering of the temperature at which the densification starts, as for GSTO, and to extend the early stage occurring before the constant rate sintering takes place. At the same time, the duration of the densification stage with decreasing rate became shorter and associated to a lower sintering efficiency. Regarding the relative density (Figure 3B), VICH and SOPO were capable of achieving the highest values (0.93–0.97, respectively), while the maximum relative density for the other bodies were lower, and particularly low for GSTO (0.87).

The sintering kinetics as calculated for all the batches from the isothermal curves (as described in Figure S3) are reported in Table S2 and Figure S4. The kinetics are different depending on the stage considered. Generally speaking, porcelain stoneware batches have a faster sintering rate than porcelains at all the stages. The kinetics are quite slow during the early stage, accelerating as the sintering reaches the constant phase (with a significant increase for GPOR) and then slow down again in the final decreasing stage.

### 3.2. Phase Composition of the Fired Bodies

**Figure 4:**
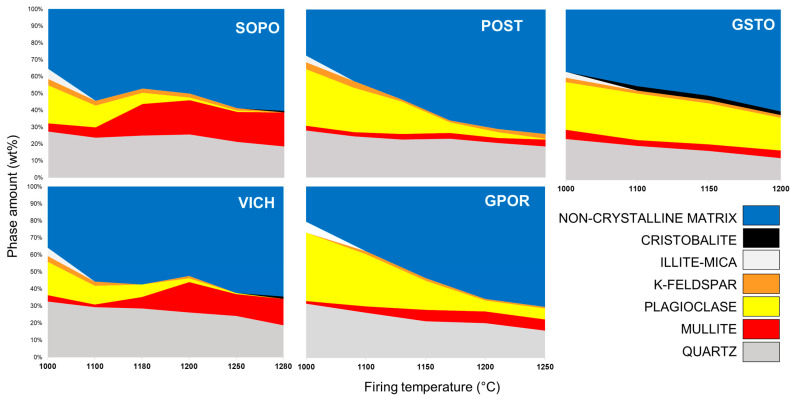
Mineralogical phase composition as a function of firing temperature.

Mineralogical phase transformations with temperature for the five ceramic batches are summarized in Figure 4 and Table S3. After firing at 1000 °C, all bodies are composed of some residual amount of illite and K-feldspar and a more consistent amount of plagioclase and quartz. Furthermore, neoformation phases (i.e., mullite, and in some cases a small amount of cristobalite) and a non-crystalline matrix are observed. Significant variations are detected with increasing temperature.

*POST-GPOR*. As previously observed [23], plagioclase—the main ingredient of porcelain stoneware—progressively melts with temperature. At 1000 °C, its residual fraction is ~70% of the initial content, and at 1250 °C it drops to 14% of the initial value and almost none for GPOR and POST, respectively. Likewise, but at a slower rate, quartz partially melts with temperature, and, even at 1250 °C, retains 64% (POST) and 50% (GPOR) of its initial content. After its formation at about 1000 °C, mullite content slightly increases with temperature mainly for GPOR (up to ~7 wt%). The non-crystalline matrix, formed after the breakdown of clay minerals and feldspars, grows rapidly with temperature, reaching the highest amount of the samples set (70–74 wt%).

*GSTO*. In this batch, plagioclase content does not vary substantially (~75% of its initial amount is retained), whilst quartz dissolves more rapidly than in the other batches (its residual fraction is 50% at 1200 °C). Similar features were already observed in porcelain stoneware bodies containing an increasing amount of glassy waste [29], and for which a relationship between the amount of waste glass and residual quartz was highlighted: namely, the higher the glass addition, the lower the quantity of quartz in the fired bodies. Moreover, the introduction of alternative fluxes [29,31,39] led to the occurrence of more feldspars at the end of the firing with respect to a waste-free body. At temperatures higher than 1100 °C, cristobalite stabilizes with a content >2 wt%, while the formation of the non-crystalline matrix occurs at a slower rate and in lower amount (~60 wt%) than for *classic* porcelain stoneware POST and GPOR.

*VICH and SOPO*. For these batches, plagioclase melts quickly up to 1200 °C, with a residual fraction of 8–10% of the starting amount, not preserved at the highest temperatures. Quartz partially dissolves above 1200 °C, but with a slower rate than porcelain stoneware (residual fraction of 70–72% at 1250 °C and 58–63% at 1280 °C). Abundant mullite (up to 20 wt%) stabilizes in the 1100–1200 °C range, then, it undergoes some dissolution–precipitation at the highest temperatures. Due to the latter mechanism, the amount of non-crystalline matrix tends to fluctuate with temperature [40,41], until the maximum content is reached (60–65 wt%).

**Figure 5:**
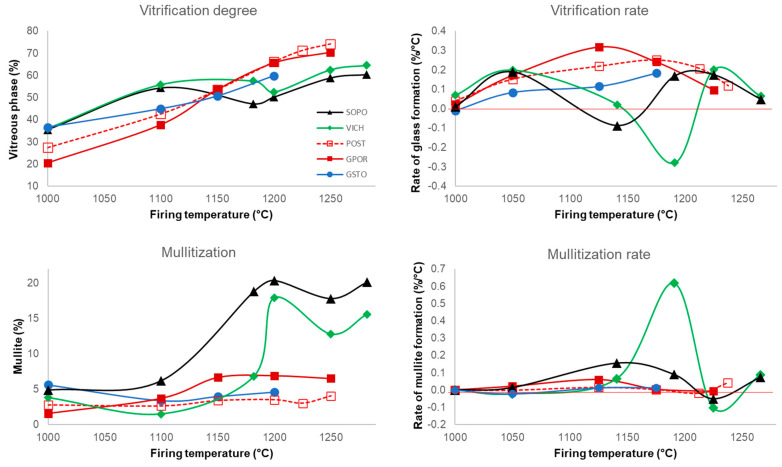
Amount of vitreous phase and vitrification rates (above); amount of mullite and mullitization rate (below) vs. firing temperature.

The peculiar variation of the non-crystalline phase with temperature observed for porcelains differs to that of porcelain stoneware bodies. As shown in detail in Figure 5, GPOR, POST and GSTO are characterized by an increasing vitrification degree with temperature, but they differ for the rate of glass formation, which is higher in GPOR before 1150 °C, due to the massive melting of feldspars. Then, a conspicuous crystallization of mullite (the highest among porcelain stoneware) decreases the rate.

The case of SOPO and VICH is more complex. After a starting high vitrification degree (even higher than porcelain stoneware) that corresponds to a sudden feldspar melting in the 1000–1100 °C interval, there is an abrupt decreasing in the rate for SOPO, due to the strong mullite crystallization in the 1100–1180 °C range, passing from 6 to 19 wt%. VICH shows the same behavior in a subsequent temperature range (1180–1200 °C) with a drop of the vitrification rate due to increasing of mullite from 7 to 18 wt%. This arrest in the vitrification path is partially damped above these temperatures, as a partial dissolution (followed by re-precipitation) of mullite takes place, along with quartz involvement in melting reactions.

### 3.3. Microstructural Evolution

**Figure 6:**
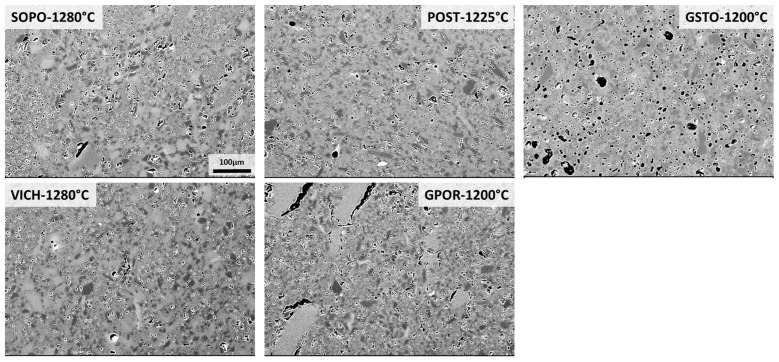
SEM micrographs of bodies at the optimal firing temperature (scale bar = 100 µm).

The above-described phase transformations entail a microstructural rearrangement of the ceramic bodies during firing. The microstructure of these bodies at the optimal firing temperature was investigated through FEG-SEM, and the resulting images from backscattered electrons are reported in Figure 6. All the batches show a compact microstructure with relics of quartz (dark gray) for porcelain and quartz and feldspars crystals for porcelain stoneware, i.e., the so-called “skeleton”. This skeleton is coupled with rounded isolated pores (from a few up to ~20 μm in diameter) and irregularly shaped pores. Mullite crystals formed during firing are tiny, usually a few micrometers in size and were investigated on the etched surfaces, and discussed below. Crystals and pores are dispersed in an abundant non-crystalline matrix (a liquid phase at high temperature), which promotes the sintering process by wetting the mineral particles and reducing the voids, thereby increasing the bulk density of the ceramic body. These microstructural features are typical of sintered porcelain [42] and porcelain stoneware [23,31].

In detail, SOPO, VICH and POST show a lower pores volume, also smaller in size, than that of the other samples, corresponding to a higher bulk density (2.39–2.44 g/cm^3^) and lower closed porosity (Figure 6, Table S1). GSTO has the lowest degree of densification (2.31 g/cm^3^) and presents a bimodal distribution of porosity: relatively large, closed pores (~20–30 μm in diameter) together with a series of small pores, smaller than 10 μm. The irregularly shaped pores of larger dimensions that can be observed in GPOR, are presumably due to powder compaction defects [39].

**Figure 7:**
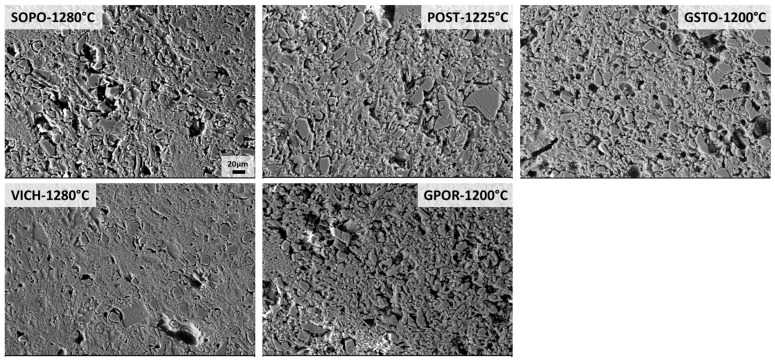
SEM micrographs of densified bodies after HF etching (scale bar = 20 µm).

**Figure 8:**
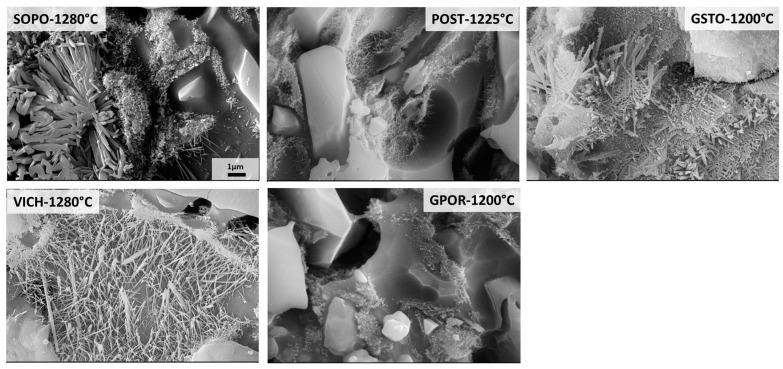
SEM micrographs after HF etching for mullite observations (scale bar = 1 µm).

**Figure 9:**
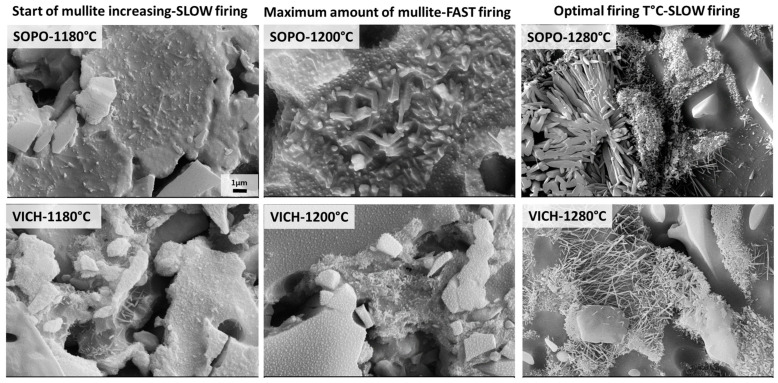
Mullite morphology of porcelains by SEM micrographs after HF etching (scale bar = 1 µm).

In order to investigate the phase morphology, the sample’s polished surfaces were etched with HF, and secondary electron images (SEI) were collected by FEG-SEM (Figure 7 and Figure 8). Moreover, in addition to the gresification temperature, the morphology evolution of the mullite for porcelain bodies was also investigated at 1180 °C, i.e., when a large amount of mullite begins to crystallize, and at 1200 °C, when the maximum amount of mullite is recorded (Figure 9).

The etched microstructures of the bodies fired at their gresification temperature are reported in Figure 7. All of them show a microstructure with crystalline particles held together by a finer matrix consisting of mullite crystals and a glassy phase (etched). However, some differences related to the residual phases arise. POST, GPOR and GSTO are characterized by coarse and angular grains of quartz and especially feldspars, these latter still present in significant amount (5%, 7% and 21 wt%, respectively). On the other hand, feldspars are totally decomposed in SOPO and VICH at 1280 °C (their optimal firing level) and the bodies are just comprised of mullite crystals, quartz grains and a glassy phase.

The investigation of mullite was performed at higher magnification, as reported in Figure 8. Different features allow to discriminate SOPO-VICH from POST-GPOR-GSTO, but also between porcelain stoneware bodies. Following the classification proposed by Iqbal and Lee [42,43,44] and extensively adopted in other researches [5,40,45,46,47], it is possible to identify in GPOR and POST the presence of only Type I primary mullite, with small size (always <1 µm) and cuboidal or elongated shape (with low aspect ratio ≤3:1). This type of mullite derives directly from clay relicts and was observed in studies of mullite formation from thermal decomposition of pure kaolinite [48,49]. On the other hand, GSTO also shows the presence of Type II secondary mullite, as grains of ~2 µm, with a higher aspect ratio (on average 8.5:1). Type II mullite originates from regions in which feldspars are well-mixed with kaolinitic clay or where fluxes penetrate clay agglomerates to form needle-shaped crystals, termed secondary mullite since they form later in the firing process [44]. The increase in aspect ratio from Type I to Type II mullite is due to a decrease in the viscosity of the liquid phase. Indeed, the melting of fluxes forms a liquid phase with a lower viscosity than that around pure clay agglomerates, and mullite crystals of neoformation can grow up more easily [42,44,45]. In GSTO, the formation of Type II secondary mullite was possible since the partial replacement of Na-feldspar with soda–lime glass significantly accelerated the sintering process, with an early formation of a non-crystalline phase, which in turn reacted with clay minerals to form secondary mullite [46]. Indeed, at 1000 °C, GSTO shows a content of non-crystalline phase higher than that of other porcelain stoneware bodies (37 wt% vs. 20–27 wt% of POST and GPOR, respectively) which allowed to accelerate the mullite growth. In Figure 8, it can also be seen Type II crystals adjacent to regions of Type I mullite (GSTO). As suggested by Martin Marquez and co-workers [45], the co-presence of both types of mullite may indicate that primary crystals, when formed at the surface of clay agglomerates, could grow out and transform into secondary mullite if close to a matrix with a lower viscosity. This result also agrees with the observation of Lundin [50], who suggested that mullite in clay relicts serves as a seed for the crystallization of mullite needles in feldspar relicts.

Porcelain bodies are clearly distinguishable from porcelain stoneware, specifically for the mullite morphology. In detail, the micrograph of the SOPO batch is representative of a series of typical porcelain features, such as quartz surrounded by a commonly termed “amorphous silica solution rim” (from dissolution of quartz grain edges at high temperature), as well as the contemporaneous presence of different types of mullite [42,43,44,45]. In fact, Type I primary mullite (small size, ~0.1–0.5 µm, and low AR, 1:1–3:1) is coupled with larger needles, which exhibit a considerable growth both longitudinally and axially, with AR∼4:1–7:1. By considering the notation proposed by Iqbal and Lee [42,43,44], these crystals should be classified as secondary Type II mullite. However, a detailed inspection of these needles allowed revealing an internal hole along their longitudinal axis (left edge of the image). This morphological feature was already detected in porcelain stoneware and porcelain bodies and attributed to the co-growth of needle-like crystals of smaller width [5,45,47]. It seems that Type III secondary mullite fibres, with aspect ratio >30:1, join together originating “clusters or packs of needles”. This cluster is formed by the rapid union of several needles, without the liquid phase receding; hence, the liquid phase remains trapped within the cluster, inducing the hole when the surface of polished samples is etched for SEM observation [5]. Along with this less common (but already documented in literature) occurrence of a Type III secondary mullite, in SOPO the “classical” Type III with an aspect ratio ~30:1, is also detected. Recent studies indicate that this mullite type develops when the viscosity of the ceramic matrix is low, as in the case of a homogeneous mixture of small size particles of quartz, feldspar and clay minerals [5,45,47]. At any rate, what was observed here seems to better fit a different situation. In fact, elongated Type III crystals are clearly visible in the micrograph of VICH body, together with Type II mullite. In this case, Type III occurs in a glass-rich region, where a feldspar grain (15 µm diameter) was present in the original batch, as in the literature case documented by Lee and co-workers [44]. This low viscosity glass pond, deriving from feldspar melting, allowed the growth of mullite needles up to the highest aspect ratio. Moreover, this pond is surrounded by Type I primary mullite, which reflects the distribution of the clayey matrix in the green body.

The evolution in shape and size of mullite in porcelain bodies during firing is displayed in Figure 9. Although there is a tiny variation in content (i.e., 19–20 wt% in the 1180–1280 °C range, as detected by the QPA-XRPD analysis), the microstructure of SOPO clearly shows that mullite crystals are experiencing important morphological changes. Conversely, in the same temperature range, VICH is more affected by dissolution–precipitation phenomena of mullite, even if the amount variation of this phase seems not correlated with its morphology. For both porcelain batches, the micrographs at 1180 °C (when a significant increase of mullite takes place) and 1200 °C (when the maximum amount is reached) reveal the presence of Type I primary mullite, even if slightly bigger and thicker in SOPO than in VICH. When the highest temperature is reached and feldspars melt completely, the growth of secondary mullite occurred (both types II and III). It is also worth noting that the firing cycle of porcelain bodies at 1280 °C was performed with a slow cycle of 24 h, which allows for crystal growth, since it is very dependent on the heating rate [45,51]. The variation in size and aspect ratio from Type I to Type III mullite is critical as both strength and toughness of the porcelain increases the more the variation in the aspect ratio is marked [44]. In fact, it has been proved that mullite content affects the mechanical properties of porcelain, being the bending strength directly associated to the aspect ratio of secondary mullite, related to the interlocking of the fine mullite needles [5,45,47]. Moreover, it was observed that the increase in bending strength reaches maximum values when mullite needles join together and give rise to clusters [47], as those detected in SOPO.

### 3.4. Composition and Physical Properties of the Vitreous Phase

**Figure 10:**
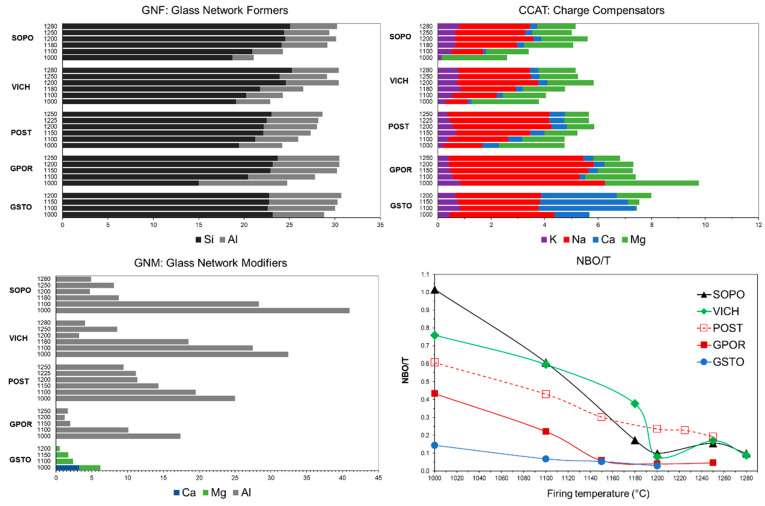
Pseudo-structural parameters of non-crystalline matrix vs. firing temperature.

The non-crystalline matrix progressively changes its composition with temperature during firing due to a complex set of reactions involving residual and newly formed crystalline phases [52]. The resulting melt composition can be better described in terms of its pseudo-structural key parameters, i.e., GNF, GNM, CCAT, and NBO/T (as defined in Section 2, and displayed in Figure 10).

In aluminosilicate-based systems, as those under examination, GNF are Si^4+^ and Al^3+^ (the latter if charge compensated by alkali and alkaline earths) [23,29,31,53]. With the exception of the lowest sintering temperature (1000 °C), when the persistence of illite indicates that the process of vitreous phase formation is still in an early stage, the GNF content always falls between 25–30 mol%. The decomposition of clay minerals and plagioclase, make soon available a certain amount of Si and Al acting as network formers. Then, the GNF content increases with the temperature, reflecting the quartz melting and the complex dissolution/precipitation behavior of mullite. The amount of GNF in all the thermal range is of 15–25 mol% for Si (always the major GNF) and 2–10 mol% for Al.

**Figure 11:**
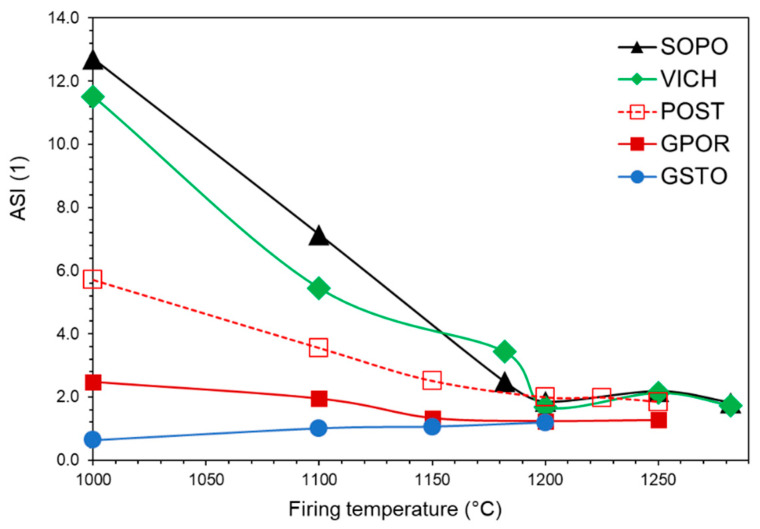
Alumina saturation index (ASI) vs. firing temperature.

Alkali and alkaline earths playing the role of charge compensators for Al^3+^ ions in tetrahedral coordination (CCAT), are present in different proportions in the melts. From one side, POST, VICH and SOPO are characterised by CCAT <6 mol%, with a predominance of Mg at the lower temperatures (due to the breakdown of clay minerals), then gradually reached up by Na as feldspars melt. On the other side, GPOR and GSTO have higher amounts of CCAT (on average >7.5 mol%), mainly Na in GPOR, due to the higher starting content of sodic feldspar, and mainly Na and Ca in GSTO, derived from the silica–lime–soda glass. Since K, Na, Ca and Mg are fully engaged as CCAT in the melts of SOPO, VICH, POST and GPOR, only Al can play as glass network modifier (GNM). Al^3+^ in excess of CCAT, which cannot be compensated, turns its behaviour from network former to modifier, by breaking GNF–O bonds and leading to a depolymerization of the melt network. In the 1150–1280 °C range, its content is quite high in porcelains (on average 8 ± 5 mol%), but largely variable in porcelain stoneware: low in GPOR (~2 mol%) and very high in POST (on average 12 ± 2 mol%). Extremely high values of Al as GNM in the early stage of sintering (1000–1100 °C range) derive from the predominant mechanism of clay minerals breakdown. This mechanism seems to be confirmed by the variation of ASI with temperature (Figure 11). In the first temperature range, the amount of Al_2_O_3_ in the melt not provided by feldspars is very high in porcelains (i.e., ASI 11.5–12.7), due to the large content of clay minerals in the starting batches; while it is lower in porcelain stoneware (POST > GPOR > GSTO). ASI values scale exactly with Al^3+^ as GNM: the higher the ASI, the higher the fraction of Al that can play as modifier. Going on with the temperature, a complex phase evolution takes place, influencing the role of Al^3+^. The ASI decreasing with temperature corresponds to the involvement of feldspars in melt formation. On the other hand, the decreasing of Al^3+^ as GNM with increasing temperature (especially in porcelain), reflects a conspicuous crystallization of mullite, which occurred from 1180 °C onwards, while the subsequent fluctuation mirrors the stability of mullite in contact with the melt (Figs. 10–11). GSTO behaves differently concerning GNM: some Ca and mostly Mg exceeded the stoichiometric ratio with Al, and can serve as modifiers, even if present in a small amount (≤6 mol%) if compared with GNM of the other melts.

The NBO/T parameter allows following the structural evolution of the melts (Figure 10). GSTO has very low NBO/T values, slightly decreasing with temperature (0.14 → 0.03), i.e., the highest degree of polymerization of the set. GPOR shows a similar behaviour, particularly from 1150 °C onwards, when the significant precipitation of mullite (up to 6.9 wt%) decreases the content of Al^3+^ as GNM, and the dissolution of quartz increases the content of Si as GNF, making the melt more polymerized (NBO/T ≤ 0.06). Due to the lower formation of mullite (≤4 wt%), GNM keeps quite high in POST, corresponding to less polymerized melts (NBO/T 0.61 → 0.19), with an intermediate trend between porcelain stoneware and porcelain. SOPO and VICH, which have the most de-polymerized melts at the lowest temperatures, then exhibit the most significant drop in NBO/T values (due to the simultaneous mullite crystallization and quartz melting), which yields at 1200 °C quite polymerized melts (NBO/T 0.08–0.10). Subsequent fluctuations are related to the dissolution/precipitation of mullite, which acts as Al^3+^ buffer in the ceramic bodies.

**Figure 12:**
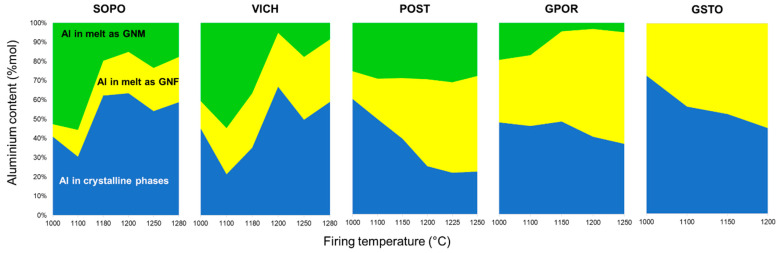
Speciation of Al in the bodies as a function of firing temperature.

The inspection of the previous pseudo-structural parameters highlights the importance of a detailed investigation of Al^3+^ speciation, i.e., its partition between crystalline phases and melt in ceramic bodies (Figure 12). For the GSTO and GPOR, Al^3+^ in melt mainly acts as GNF as it occurs almost exclusively as tetrahedrally coordinated. Only a small fraction of Al^3+^ in the GPOR melt acts as GNM. This fraction decreases with temperature, being related to the concomitant mullite crystallization. The fraction of Al^3+^ hosted at tetrahedral and octahedral sites of GSTO and GPOR crystalline phases shows similar trends, but related to different phenomena. The aluminum fraction in the GSTO crystalline phases gradually decreases because of a feldspars slow dissolution. The aluminum fraction in the GPOR crystalline phases decreases even more slowly, since the dissolution of feldspars is in some way counterbalanced by the precipitation of mullite. In POST, the melting of feldspars is not fully compensated by the mullite formation, therefore, as the temperature increases, Al^3+^ is incorporated into the melt, where it behaves as GNF. The fraction of Al^3+^ which acts as GNM keeps constant throughout the investigated thermal interval. Porcelains are characterized by less regular trends that reflect the evolution of the crystalline phases with temperature (i.e., melting of feldspars up to 1180–1200 °C and the subsequent crystallization/dissolution of mullite up to 1280 °C). These phases actually represent a repository of aluminium that buffers the release in the melt depending on their stability during firing. As previously discussed (see also Figure 10), the role of Al^3+^ in the melts of SOPO and VICH is mainly that of a network modifier at the lowest temperatures, then to decrease in correspondence with the formation of mullite.

**Figure 13:**
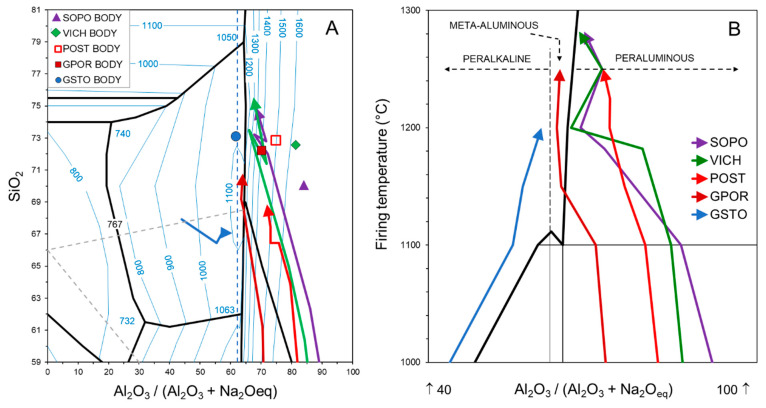
Starting chemical composition of the bodies (**A**) and trends of melts composition in the ternary diagram (**A**,**B**).

The variation of melts’ chemical composition, compared to that of the green bodies, is summarized in the SiO_2_–Al_2_O_3_–Na_2_O ternary phase diagrams of Figure 13 (for the diagram construction, see Conte et al. [29]).

Porcelains have strongly peraluminous bodies, which give rise to strongly peraluminous melts at the lower temperatures, as testified by the high presence of Al^3+^ as GNM. Then, such melts move rapidly towards the silica vertex and the main cotectic line of the Na_2_O-Al_2_O_3_-SiO_2_ diagram, due to the simultaneous quartz dissolution and mullite crystallization. As discussed above, trend fluctuations above 1180 °C are those derived from the complex behavior of mullite. POST also has a strongly peraluminous body and, indeed, the trend defined by the melt chemical variation approaches that of porcelain, with a composition more and more enriched in silica due to the quartz melting. However, it keeps a frankly peraluminous composition also at the highest temperatures, due to the scarce mullite formation. Otherwise, the frankly peraluminous GPOR body produces a slightly peraluminous melt at low temperatures, which moves quickly to the cotectic line, reaching the meta-aluminous field. The glass-bearing stoneware has a meta-aluminous body, evolving in melts which keep their composition in the peralkaline field, moving towards the cotectic line by increasing temperature and finally approaching the meta-aluminous line again. Indeed, GSTO is quite close to the feldspar stoichiometry and this justifies the stability of plagioclase at all firing conditions (Figure 4, Table S3). At temperature >1150 °C, GPOR is the closest to a thermodynamic equilibrium, as the melt composition evolves along the liquidus surface near the isotherm of 1200 °C.

**Figure 14:**
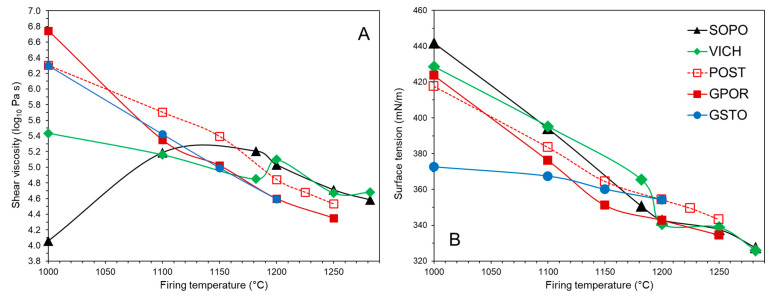
Shear viscosity (**A**) and gas–liquid surface (**B**) of the melts vs. firing temperature.

The physical properties of the melt, such as shear viscosity and gas–liquid surface tension, play a key role during the viscous flow sintering. They are shown in Figure 14 and Table S4, as predicted for the various firing temperatures. 

The melt structure, as described above, directly affects the physical properties at high temperature. Specifically, the more polymerised melts (low NBO/T) of GSTO, GPOR and POST are initially characterised by a higher viscosity with respect to the porcelain; then, with increasing temperature, their viscosity linearly decreases, as already observed for other porcelain stoneware [23]. The starting higher viscosity of GPOR melt agrees with its metaluminous composition (Figure 13). In fact, as recorded for natural aluminosilicate melts, shear viscosity peaks at the metaluminous join [28]. On the other hand, VICH and especially SOPO melts are characterised by a more complex trend. The higher degree of de-polymerization in the lowest thermal range (high NBO/T) reflects in a lower viscosity, which oscillates with temperature because of the mineralogical phase evolution of the above. In SOPO, the melt viscosity rapidly increases up to 1100 °C, and keeps constant during the mullite formation (which subtracted Al^3+^ as GNM from the melt) and the parallel dissolution of quartz (which increases the concentration of GNF in the melt). At higher temperature, the viscosity decreases as expected, as only a small fraction of quartz dissolves. The VICH melt, after a drop in viscosity up to 1180 °C, follows substantially the same behaviour as SOPO. Focusing the attention on the melt viscosity at the optimal firing temperature, similar values can be observed for each ceramic body. That is, the melt viscosity is ~4.6 to 4.7 Log_10_ Pa⋅s with minimal variations from GSTO and GPOR at 1200 °C, POST at 1225 °C, up to SOPO and VICH at 1280 °C (Table S4). This means that, at the optimal firing temperature, the shear viscosity for each batch is comparable with values recorded for other porcelain stoneware bodies: sodic 4.9 Log_10_ Pa⋅s [23], containing strong fluxes 4.4–4.8 Log_10_ Pa⋅s [31], based on K- feldspars or sericite 4.5–4.7 Log_10_ Pa·s [53], as well as standard and hard porcelain 4.4–4.7 Log_10_ Pa⋅s [36]. Therefore, it seems that the viscous flow sintering of silicate ceramics takes place in a critical window of melt viscosity, irrespective of the firing temperature.

The gas–liquid surface tension of melts sharply drops as the temperature increases, with similar trends for all the series but for GSTO. The latter displays a starting value lower than the other batches (367 vs. 376–395 mN⋅m^−1^ at 1100 °C) coupled with the lowest rate of surface tension decreasing with temperature. If compared at the temperature of optimal firing, the gas–liquid surface tension of the melts is quite similar for GPOR, POST and GSTO (343–354 mN⋅m^−1^) and comparable with values estimated for sodic batches ~340 mN⋅m^−1^ [23], bodies containing glassy waste 342–359 mN⋅m^−1^ [29] or strong fluxes 341–369 mN⋅m^−1^ [31]. VICH and SOPO melts have the lowest surface tension (326–327 mN⋅m^−1^), not far from that recorded for porcelain by Conte et al. [36].

**Figure 15:**
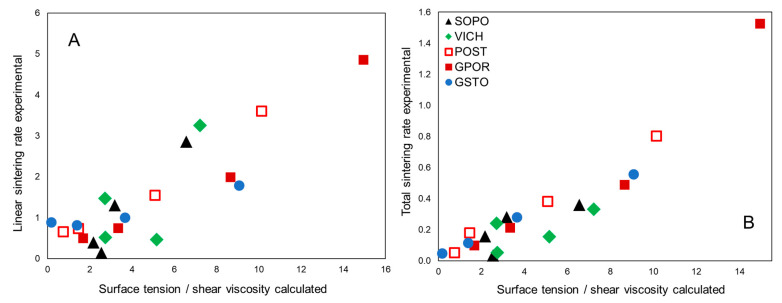
Sintering ratio vs. experimental sintering linear rates (**A**) and vs. experimental sintering total rates (**B**). Experimental uncertainty is within the symbol size.

The surface tension to shear viscosity ratio is the fundamental term in the equations used to describe the viscous flow sintering kinetics in glasses, as those of the Frenkel and Mackenzie–Shuttleworth models [54]: the higher the ratio, the faster the expected densification rate. This parameter, already successfully used to describe the sintering kinetics in porcelain stoneware bodies [23,29], is here calculated and contrasted with the experimental linear and total rates, as derived from the HSM isothermal tests (Figure 15).

There is a general agreement between experimental and calculated data, especially for porcelain stoneware (GPOR, POST and GSTO), considering the total sintering rate (Figure 15B). This is because the linear stage accounts for just a small fraction of the sintering process (24 ± 6%, Figure 2), while the total rate allows to compare the sintering kinetics all along the process. A less obvious correlation can be observed for porcelains, whose melts have a complex evolution of viscosity and surface tension as a function of temperature. This is reflected in densification rates that fluctuate with temperature, especially for VICH, and align to the rates recorded for porcelain stoneware only between 1200 and 1250 °C.

### 3.5. Dimensional Stability at High Temperature

The physical properties of the melt are expected to play a crucial role also on the dimensional stability of the bodies at the maximum temperature, which in turn borders phenomena such as firing deformation and pyroplasticity [55,56,57]. However, in order to investigate deformation phenomena and de-sintering processes, the physical and microstructural properties of both the melt and the ceramic ware, as a bulk, have to be considered. In fact, it is known that firing deformations depend on the amount and viscosity of the liquid phase, which is responsible for flow movements at the origin of pyroplastic effects [55,58]. Nevertheless, solid particles do contrast such deformations, in a more or less effective manner in function of amount, size and shape of the crystalline compounds constituting the skeleton [57,59,60]. It can be said that the dimensional stability of the bodies at the maximum temperature is related with the bulk viscosity of the body, here called *effective viscosity* (η_eff_), which combines melt and crystals effects.

**Figure 16:**
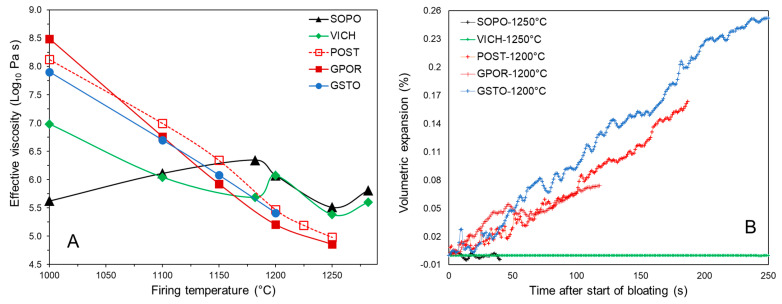
Effective viscosity vs. firing T °C (**A**) and bloating degree (**B**).

The effective viscosity of porcelain stoneware (GPOR, POST and GSTO) retraces the trend of the melt viscosity, with very high values at the lowest temperature (7.9–8.5 Log_10_ Pa⋅s at 1000 °C, Figure 16A). This is the result of the interplay of well polymerized melts, together with a high solid load, ~80 wt% of the body at 1000 °C in GPOR. The effective viscosity sharply decreases with temperature, so reflecting the simultaneous reduction in melt viscosity and amount of solid load.

Porcelains exhibit a peculiar trend of effective viscosity, which does not strictly follow that of the melt. The crystallization of secondary mullite in VICH and SOPO leads to an increase of both the solid load over 1200 °C and the mean aspect ratio, and consequently of the relative viscosity. Moreover, it must be noted there is a significant increase in the effective viscosity at the highest temperature, due to increasing of the mullite aspect ratio from Type I and II to Type III (as observed in Figure 8 and Figure 9). If compared at the optimal firing temperature, porcelain stoneware shows values of effective viscosity around 5.2 Log_10_ Pa⋅s (POST and GPOR). The lower solid load in POST at 1225 °C (~30 wt% vs. 35 wt% at 1200 °C for GPOR) is compensated by a higher melt viscosity (with a similar mean aspect ratio of crystals). GSTO at 1200 °C exhibits a higher effective viscosity of 5.41 Log_10_ Pa⋅s, due to the relevant content of the crystalline fraction, corresponding to ~40wt% of the body. Porcelains are characterised by the highest η_eff_ values (5.60–5.81 Log_10_ Pa⋅s) at 1280 °C, as a consequence of secondary mullite high aspect ratio, which strongly affects the rheological behaviour of the bulk: interlocking of fine mullite needles, as in VICH, or presence of clusters of needles, as in SOPO. 

A direct measure of the dimensional stability at high temperature is the tendency of the bodies to expand during dwell time. This is a complex phenomenon, caused by both bloating (driven by gas expansion in the closed porosity) and collapsing of the specimen under its own weight (because of a too low viscosity). The expansion rate was here investigated by isothermal runs through optical dilatometry (Figure 16B). Porcelain stoneware bodies tend to expand linearly with dwell time, once the maximum densification is reached. GSTO suffers from the highest bloating rate, coherently with the high values of closed porosity (Figure 1 and Figure 6). POST and GPOR are characterized by slower expansion rates and a lesser closed porosity as well. Vice versa, VICH and SOPO do not exhibit any significant expansion during dwell time, suggesting a very good stability at high temperatures. The differences in stability between porcelain and porcelain stoneware are well explained by the effective viscosity of the ceramic bodies reported above. In turn, the rheological behaviour of the bulk depends on the body microstructure and, particularly, on the size and shape of crystals dispersed in the melt [55,56]. Looking at porcelain stoneware in detail, however, this observation holds only for POST and GPOR that have similar bloating rates matching a very similar η_eff_ at the temperature of optimal firing. GSTO is affected by a bloating rate that exceeds what is expected from its effective viscosity. GSTO expansion can be likely explained by the “nature” of its skeleton (mainly plagioclase), and a long permanence at the maximum temperature (30 min). In fact, any dissolution of quartz during the dwell time would lead to a decrease in the solid load counterbalanced by an increasing viscosity of the liquid phase (increase in silica content) [23]. In contrast, the dissolution of feldspars (with release of alkali and alkaline earths) would lead to a de-polymerization of the melt and to a consequent drop in viscosity of the liquid phase with no counterbalancing effect, as solid load decreased as well, hence promoting the bloating.

### 3.6. Interplay of the Considered Sintering Parameters

Different behaviors emerged during the sinter-crystallization process of the five bodies with an interplay between all the considered sintering parameters, here discussed.

*Vitreous china and soft porcelain*: formulated with the same fraction of quartz and feldspar, but different kaolin/ball clay ratios. The almost double content of kaolin in SOPO, in contrast to VICH, made this body less compressible, corresponding to a lower unfired bulk density. Anyway, both reached the maximum density and water absorption <0.5 wt% at 1280 °C. Regarding the sintering mechanisms investigated by HSM tests, thanks to a predominance of the final stage of sintering (which means that a substantial part of densification took place with a decreasing rate), these bodies were able to reach the best densification efficiency among the investigated samples. The vitrification path, which governs the viscous flow sintering, is characterized by fluctuating degree and rate, due to a strong phase of de-vitrification in correspondence with massive mullite crystallization (up to ~20 wt%), followed by dissolution/precipitation phenomena, mostly in VICH. From the microstructural point of view, at their optimal firing temperature, porcelain bodies are just composed of quartz (smaller dimensions with respect to porcelain stoneware) together with mullite and glassy phase. Their peculiarity is the presence of different types of mullite: Type I primary mullite (below <0.5 µm and AR ≤2.5:1); Type II secondary mullite (dimension ~2 µm and AR ≤9:1); Type III secondary mullite (with even higher AR). Specifically, this latter type exhibits different morphologies: fibres with aspect ratio >30:1, and fibres joined together to form “clusters or packs of needles”, which strengthen the porcelain microstructure. As far as the VICH and SOPO melts are concerned, the strongly peraluminous chemical character of the early sintering stage (due to the breakdown of phyllosilicates and limited precipitation of mullite), changes rapidly moving towards the silica vertex and the main cotectic line of the Na_2_O-Al_2_O_3_-SiO_2_ diagram, thanks to the simultaneous quartz dissolution and mullite crystallization. Above 1180 °C trend fluctuations are observed, depending on the complex behavior of mullite. This change in melt composition affects its physical properties in a non-linear way as a function of temperature, also reflecting in fluctuating densification rates. Dimension stability and de-sintering process of ceramic bodies at high temperature are controlled by the effective viscosity (i.e., the ceramic bulk viscosity). At the optimal firing temperature of 1280 °C, SOPO and VICH exhibited the highest η_eff_ values of the samples set (5.6–5.8 Log_10_ Pa⋅s), not only due to an increase of the solid load for the precipitation of secondary mullite, but also due to the growth of its aspect ratio, which strongly affects the rheological behavior of the ceramic bulk. Indeed, during the isothermal runs the porcelains did not show any expansion with dwell time, indicating a very good stability at high temperatures. It can be concluded that in porcelain, the behaviour during the sintering is governed essentially by the body microstructure: depolymerized melt allows a fast sintering rate until crystallization and evolution of mullite types I–II–III increase the effective viscosity. This occurs by increasing both the solid load and aspect ratio, and by turning the liquid phase more polymerized.

*Porcelain stoneware*: the slightly diverse formulations adopted to appraise the effects of a different ball clay to kaolin ratio and a different amount and source of sodic feldspar, actually induced appreciable variations between POST and GPOR. From the technological point of view, being composed of a smaller fraction of feldspars, the optimal firing temperature of POST is higher than GPOR (1225 vs. 1200 °C). The sintering mechanisms, investigated by *in-situ* HSM tests, revealed an extent of the non-isothermal stage (representing the densification occurring during the heating ramp) higher in POST, which reflected in a lower efficiency of densification than GPOR. This lower densification degree could be just apparent, since the temperature adopted for the isothermal run of POST (1200 °C) is slightly lower than that of optimal firing. In fact, considering the technological properties of the two bodies at their gresification temperature (*ex-situ* tests), they show the same final bulk density (2.39 g/cm^3^). POST and GPOR are characterized by the highest degree of vitrification (70–74 wt%) among the batches under examination, with a maximum rate of amorphous phase formation around 1150 °C, in correspondence with a massive feldspar melting. The degree of mullitization is higher in GPOR than POST, as consequence of the higher kaolin-to-ball-clay ratio. At the optimal firing temperature, these phase transformations produced a compact microstructure, composed of a vitreous phase embedding a skeleton of quartz and feldspars, consisting of quite coarse and angular relics. Mullite is small-sized and exclusively of the primary Type I, deriving from clay minerals. The evolution of the melt chemical composition is mainly influenced by the mullite formation: in POST, which developed a lower amount of this phase, the melt remained strongly peraluminous, while in GPOR, it moved towards the meta-aluminous field, since the mullite crystallization subtracted Al^3+^ from the melt. This meta-aluminous character of GPOR melt heavily influenced the sintering kinetics, resulting in being the fastest between the bodies under investigation. Moreover, the melt became more and more rich in silica by increasing the temperature in both batches, due to the partial dissolution of quartz, leading to an ever-higher degree of melt polymerization. The physical properties of the melt, shear viscosity and surface tension, decrease almost linearly with the temperature in both the samples, as usually observed for porcelain stoneware. This trend reflected in a calculated sintering rate which linearly increased with the temperature, in agreement with the experimental data collected by HSM *in-situ* tests. At the gresification temperature, POST and GPOR showed the lowest η_eff_ values of the samples set (5.2 Log_10_ Pa⋅s), corresponding to a moderate pyroplasticity (measured as body expansion). It can be concluded that in porcelain stoneware the behaviour during sintering is governed mostly by the chemical composition of the melt (and, in turn, by its properties). In fact, it also determines the stability of quartz and mullite at high temperature, ensuring a sufficient skeleton to constrain the deformations. Actually, GPOR is the only batch with a melt that over 1150 °C approached the thermodynamic equilibrium.

*Glass-bearing stoneware*: containing 20 wt% of recycled glass cullet (soda–lime–silica) in partial substitution of Na-feldspar. GSTO showed all the technological features typical of porcelain stoneware known for batches bearing >10 wt% of SLS glass. Its densification started at the lowest temperature, greatly extending the early stage sintering, occurring before a constant rate takes place. Nevertheless, a conspicuous shortening of the decreasing stage induced the worst efficiency of densification, as testified by the lowest bulk density in the *in-situ* and *ex-situ* test, as well as by the largest closed porosity with respect to both porcelain stoneware and porcelain. The early start of the sintering process in GSTO corresponds to a high vitrification degree at lower temperature than POST and GPOR. At the same time, the vitrification rate kept almost constant with temperature in GSTO, without the increasing observed in typical porcelain stoneware, resulting in a lower vitreous phase amount at 1200 °C. This lower content of non-crystalline phase is caused by a larger residual solid load, mainly composed of plagioclase (well distinguishable in the SEM micrographs). The stability of plagioclase in GSTO after all firings is due to its meta-aluminous character, quite close to the feldspar stoichiometry. At the same time, this chemical composition did not promote the mullite formation (≤5 wt%). At any rate, even if present in small amount, both Type I primary and Type II secondary mullite occurred in GSTO. The formation of secondary mullite was possible since the partial replacement of Na-feldspar with soda–lime glass significantly increased the vitrification degree, allowing the growth of Type II crystals in glass-rich zones in contact with clay relics. The chemical composition of the melt present in GSTO remained in the peralkaline field all along the temperature range, approaching the meta-aluminous line at 1200 °C, i.e., its optimal firing temperature. This gave rise to a well polymerized melt, corresponding to a high viscosity, which linearly decreased by increasing the temperature, as observed for the other porcelain stoneware. In this sense, GSTO is characterized by a behaviour very similar to POST and GPOR, with a calculated sintering rate matching data from the HSM *in-situ* tests. At the optimal firing temperature, GSTO has an effective viscosity higher than POST and GPOR (5.4 vs. 5.2 Log_10_ Pa⋅s). This fact, in principle, should have ensured a good stability during dwell time. However, this is in contrast with experimental data that indicate significant bloating phenomena. At variance of ordinary porcelain stoneware, the solid load in GSTO is mainly composed of plagioclase, which can melt during the long dwell time of the isothermal test (30 min), leading to a change in melt chemical composition and properties. Actually, the release of alkali and alkaline-earths from the feldspar to the melt induces a decrease in viscosity, so promoting the body expansion. This is a working hypothesis, which needs further experimental validation. Overall, the behaviour of the glass-ceramic stoneware during the sintering is also governed by the chemical composition of the melt and its properties, with the stability of feldspars at high temperature playing a key role to prevent de-sintering phenomena.

## 4. Conclusions

The aim of this work was to track a clear distinction between porcelain and porcelain stoneware in terms of sintering mechanisms and kinetics, phase and microstructure evolution, and high temperature stability. Porcelain and porcelain stoneware, despite their compositional similarities, behave in a clearly different way during sintering. Such differences in firing behaviour are the convolution of a completely distinct phase evolution with effects on the composition and structure of the non-crystalline matrix.

The firing behaviour of porcelain is controlled essentially by microstructural features. It is the mullitization rate (and development of types I–II–III mullite) which creates conditions for a relatively fast sintering rate at lower temperature (depolymerized melt and lower solid load) then to contrast deformations at high temperature (enhanced effective viscosity by increasing solid load, mullite aspect ratio, and melt polymerization). 

In porcelain stoneware, the behaviour during sintering is basically governed by the physical properties of melt, which in turn depend on the chemical composition. This latter reflects the stability of quartz and mullite at high temperature. A buffering effect ensures adequate effective viscosity to counteract deformation, either by preserving a sufficient skeleton or by increasing melt viscosity if quartz is melted. The best formulation (GPOR) combines the fastest sintering rate, excellent densification, and good stability at high temperature. It is no coincidence that it is the only batch that over 1150 °C approached the thermodynamic equilibrium.

The behaviour during sintering of the glass–ceramic stoneware (i.e., containing a large amount of recycled glass) also depends on the chemical composition of the melt and its properties. At variance of ordinary porcelain stoneware, there is no buffering effect to promote the stability at high temperature since melting of feldspars shall always induce a contextual decreasing of solid load and melt viscosity. Therefore, the persistence of feldspars at high temperature plays a key role to prevent de-sintering phenomena.

The distinct role of mullite in porcelain and porcelain stoneware is of paramount importance. This entails the different effects that primary and secondary mullites have during sinter-crystallization. Mullite is crucial from the microstructural point of view in soft porcelain and vitreous china, as the growth of Type III secondary mullite with a high aspect ratio ensures a good dimensional stability at high temperature. It is important in constraining the composition of the vitreous phase in porcelain stoneware since mullite acts as a buffer of aluminium.

In conclusion, the terms *porcelain* and *porcelain stoneware* must not be confused with each other, while the term “porcelain tile” is misleading and should not be used.

## Figures and Tables

**Table 1:** Chemical and mineralogical composition of raw materials.

	Ball Clay	Kaolin	Quartz	Sodic Feldspar A	Sodic Feldspar B	Soda Lime Glass
**SiO_2_**	58.20	48.00	99.26	69.50	69.35	70.50
**TiO_2_**	1.30	0.05	0.02	0.45	0.28	0.07
**Al_2_O_3_**	26.90	37.00	0.30	18.00	18.50	3.20
**Fe_2_O_3_**	0.80	0.96	0.03	0.35	0.11	0.42
**MgO**	1.30	0.30	0.02	0.20	0.18	2.30
**CaO**	0.30	0.07	0.02	1.00	0.65	10.00
**Na_2_O**	0.20	0.10	0.10	9.50	10.30	12.10
**K_2_O**	2.00	2.00	0.04	0.40	0.28	1.00
**LoI**	8.90	12.10	0.21	0.50	0.41	0.60
**Illite**	16.5	15.6	−	−	−	−
**Kaolinite**	45.0	77.7	−	5.4	3.3	−
**Smectite**	15.5	−	−	−	−	−
**Fe-oxides**	0.9	1.1	−	0.4	0.1	−
**Quartz**	20.0	3.6	99.0	11.1	7.2	−
**Plagioclase**	0.8	0.9	−	80.5	87.3	−
**Orthoclase**	0.2	0.9	−	0.9	1.0	−
**Rutile**	1.1	0.1	−	0.5	0.3	−
**Accessories**	−	0.2	1.0	1.3	0.8	−

**Table 2:** Formulation, chemical composition and main physical properties of the raw batches.

	Unit	SOPO	VICH	POST	GPOR	GSTO
**Kaolin**	wt%	50	27	-	5	−
**Ball clay**	wt%	−	23	40	30	40
**Quartz**	wt%	25	25	15	15	13
**Sodic Feldspar A**	wt%	25	25	45	−	27
**Sodic Feldspar B**	wt%	−	−	−	50	−
**Soda–lime glass**	wt%	−	−	−	−	20
**SiO_2_**	wt%	69.56	71.97	71.97	71.66	72.53
**TiO_2_**	wt%	0.13	0.39	0.78	0.50	0.61
**Al_2_O_3_**	wt%	25.02	22.13	20.02	19.35	16.53
**Fe_2_O_3_**	wt%	0.66	0.57	0.51	0.42	0.58
**MgO**	wt%	0.86	0.89	0.66	0.70	1.07
**CaO**	wt%	0.22	0.28	0.59	0.43	2.50
**Na_2_O**	wt%	2.42	2.64	4.40	6.13	5.03
**K_2_O**	wt%	1.13	1.13	1.07	0.81	1.16
**Median particle size, d_50_**	µm	3.46	2.98	3.14	2.66	3.41
**Loss on ignition, LoI**	% wt.	6.21	4.93	3.08	2.97	3.25
**Specific weight of raw body, SW**	g·cm^−3^	2.582	2.634	2.604	2.603	2.603
**Bulk density of pressed body, BD**	g·cm^−3^	1.851	1.979	1.996	1.846	1.997
**Relative density of pressed body, RD = BD/SW**	1	0.717	0.751	0.766	0.709	0.767

## Data Availability

Not applicable.

## Supplementary Materials

The following supporting information can be downloaded at: https://www.mdpi.com/article/10.3390/ma16010171/s1, Figure S1. Particle size distribution of batches. Table S1. Technological properties of fired products: firing shrinkage (FS), water absorption (WA), bulk density (BD), specific weight of powders (SW), open porosity (OP), closed porosity (CP), total porosity (TP), and lasting of the firing cycle (CYCLE). (*e.u.*: experimental uncertainty). Figure S2. Rietveld refinement plot of sample VICH 1200 °C. The experimental data are indicated by plus signs, the calculated pattern is the continuous line and the lower curve is the weighted difference between the calculated and observed patterns. The vertical tick marks show the allowed reflections for the crystalline phases present in the sample: black for corundum, red for quartz, blue for plagioclase, green for orthoclase and yellow for mullite. Figure S3: The curve describes the linear shrinkage as a function of firing time during the isothermal run. Different stages of sintering are pointed out, with the relative turning points. Since the mechanisms governing the viscous flow sintering are known, it is possible to describe the process through the parameterization of the firing curve. At temperatures around 1000 °C, a viscous mass transport begins with a given shrinkage (from P1 to P2). This early densification occurs during the heating ramp and is promoted by the flow of the neo-formed liquid phase. Once the sample reaches the maximum firing temperature, the sintering enters the isothermal stage (from P2 to P4), with a first linear rate (from P2 to P3) and then a decreasing rate (the final densification from P3 to P4). Once the maximum density is reached, an inversion of the sintering process may take place, leading to a more or less accentuated expansion, with a bloating quantified from P4 to P5. (RD = relative density, H = height of the specimen and t = time at the point P*X*, with *X*: 1–5). Table S2. Firing behavior of ceramic batches in the HSM tests. Figure S4. Sintering kinetics (min^−1^) in the different stages of the process. Table S3. Phase composition (% weight) of the bodies fired at different maximum temperatures. Table S4. Melt physical properties at high temperature.
