# Supplementary material for: Porcelain versus Porcelain Stoneware: So Close, So Different. Sintering Kinetics, Phase Evolution, and Vitrification Paths
Source: Materials (Basel). 2022 Dec 24;16(1):171. doi: 10.3390/ma16010171 (PMC9821833; doi:10.3390/ma16010171)
Supplement: Supplementary file 1 [file materials-16-00171-s001.zip › materials-2104633-supplementary.pdf]

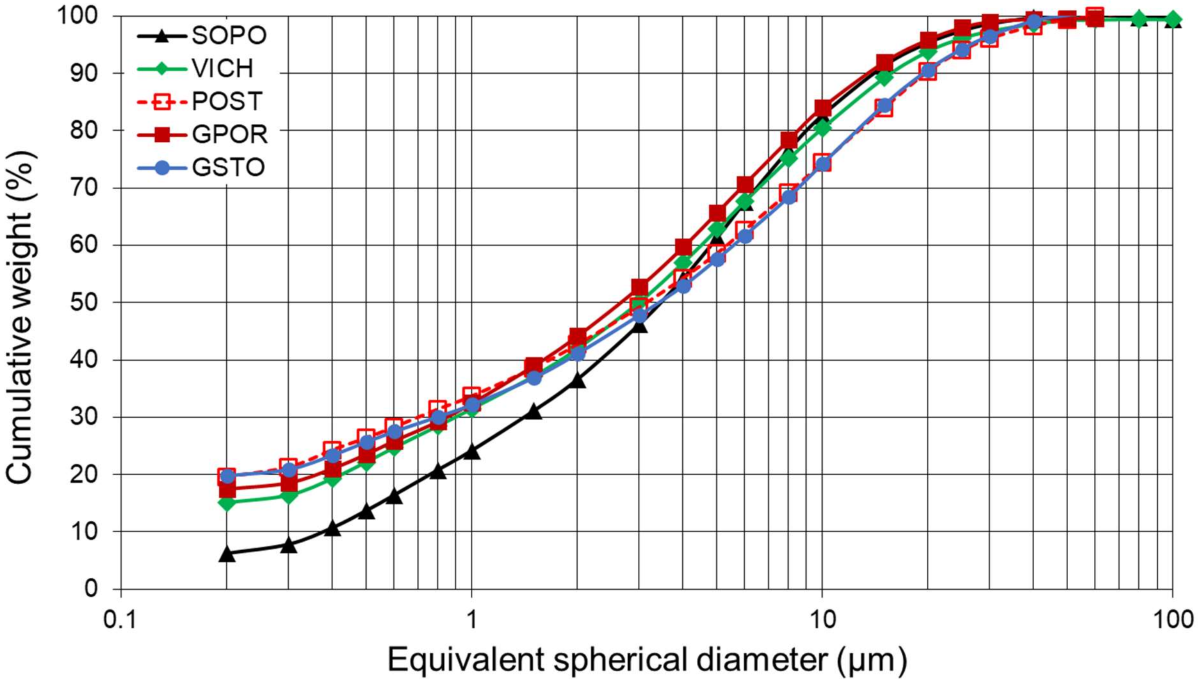

Figure S1. Particle size distribution of batches.

Table S1. Technological properties of fired products: firing shrinkage (FS), water absorption (WA), bulk density (BD), specific weight of powders (SW), open porosity (OP), closed porosity (CP), total porosity (TP), and lasting of the firing cycle (CYCLE). (*e.u.*: experimental uncertainty).

	T °C	FS %	WA %	BD g/cm³	SW g/cm³	OP %	CP %	TP %	CYCLE h
e.u.	-	*0.01*	*0.01*	*0.005*	*0.005*	*0.01*	*0.01*	*0.1*	-
**SOPO**	1000	-0.63	18.43	1.770	2.654	32.61	0.71	33.32	1
	1100	0.11	15.98	1.848	2.645	29.52	0.60	30.12	1
	1180	3.94	9.92	2.053	2.603	20.37	0.77	21.14	24
	1200	4.79	7.86	2.125	2.583	16.71	1.02	17.73	1
	1250	6.91	3.96	2.320	2.579	9.18	0.86	10.04	1
	1280	9.60	0.13	2.443	2.543	0.31	3.61	3.92	24
	1330	8.33	0.00	2.412	2.513	0.00	4.02	4.02	24
**VICH**	1000	-0.53	13.95	1.935	2.654	27.00	0.08	27.08	1
	1100	0.66	12.06	1.993	2.639	24.03	0.46	24.50	1
	1180	4.32	5.65	2.243	2.580	12.67	0.42	13.09	24
	1200	5.00	3.95	2.292	2.558	9.05	1.35	10.40	1
	1250	6.22	1.29	2.396	2.549	3.08	2.91	5.99	1
	1280	7.51	0.43	2.430	2.521	1.04	2.56	3.60	24
	1330	3.57	0.00	2.135	2.503	0.00	14.69	14.69	24
**POST**	1000	-0.52	12.65	1.972	2.654	24.94	0.77	25.71	1
	1100	0.97	10.51	2.043	2.622	21.47	0.60	22.07	1
	1125	1.62	9.24	2.090	2.614	19.31	0.71	20.03	1
	1150	3.23	6.24	2.196	2.605	13.71	2.01	15.72	1
	1175	4.49	3.53	2.304	2.529	8.13	0.77	8.90	1
	1200	5.15	1.87	2.347	2.512	4.38	2.21	6.60	1
	1225	5.25	0.42	2.389	2.483	1.00	2.79	3.79	1
	1250	4.97	0.32	2.358	2.457	0.99	3.03	4.02	1
**GPOR**	1000	-0.57	15.29	1.864	2.620	28.50	0.36	28.85	1
	1100	0.87	12.80	1.947	2.600	24.92	0.20	25.11	1
	1150	3.50	8.74	2.091	2.570	18.28	0.34	18.62	1
	1200	6.98	0.27	2.390	2.503	0.66	3.87	4.53	1
	1250	6.56	0.16	2.324	2.485	0.38	6.07	6.45	1
**GSTO**	1000	0.57	9.88	2.036	2.575	20.12	0.85	20.96	1
	1100	2.16	6.87	2.143	2.568	14.72	1.81	16.53	1
	1125	2.42	6.18	2.169	2.542	13.40	1.27	14.67	1
	1150	2.96	4.96	2.207	2.529	10.95	1.78	12.73	1
	1175	3.41	3.30	2.251	2.508	7.43	2.82	10.25	1
	1200	4.10	0.24	2.306	2.489	0.56	6.79	7.35	1
	1225	4.29	0.00	2.328	2.499	0.00	6.84	6.84	1

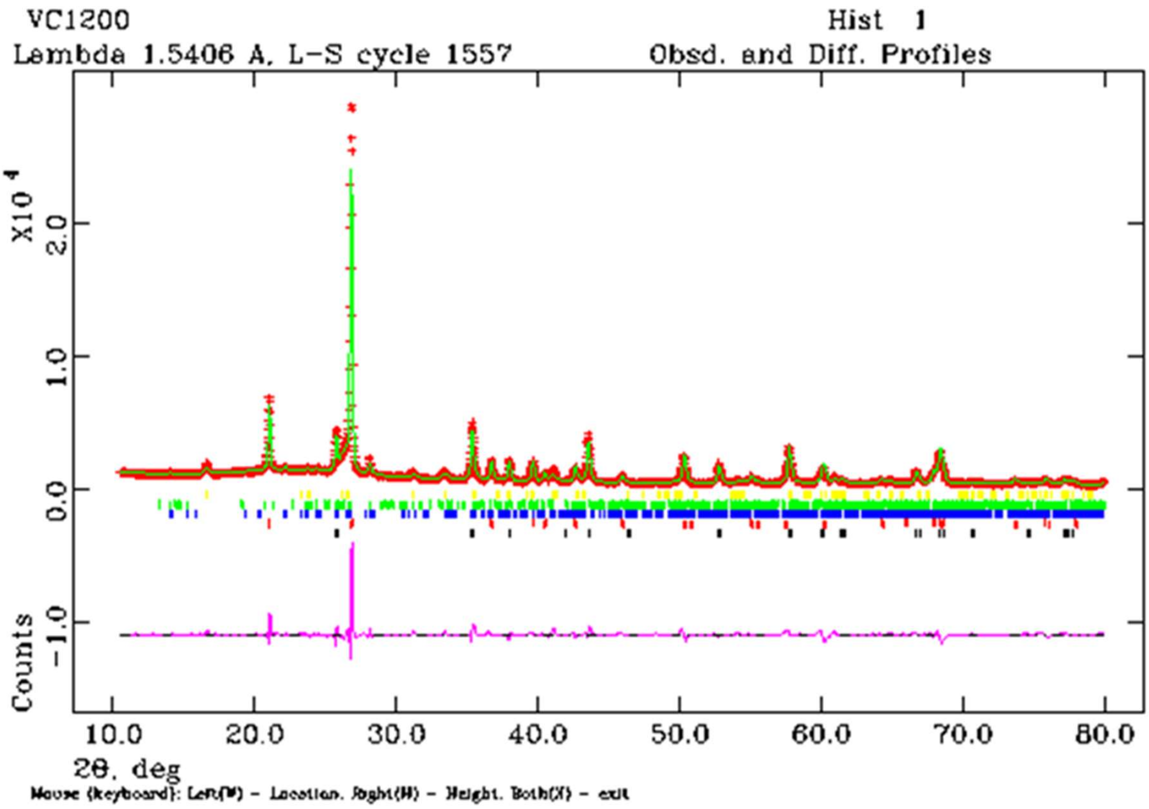

Figure S2. Rietveld refinement plot of sample VICH 1200 °C. The experimental data are indicated by plus signs, the calculated pattern is the continuous line and the lower curve is the weighted difference between the calculated and observed patterns. The vertical tick marks show the allowed reflections for the crystalline phases present in the sample: black for corundum, red for quartz, blue for plagioclase, green for orthoclase and yellow for mullite.

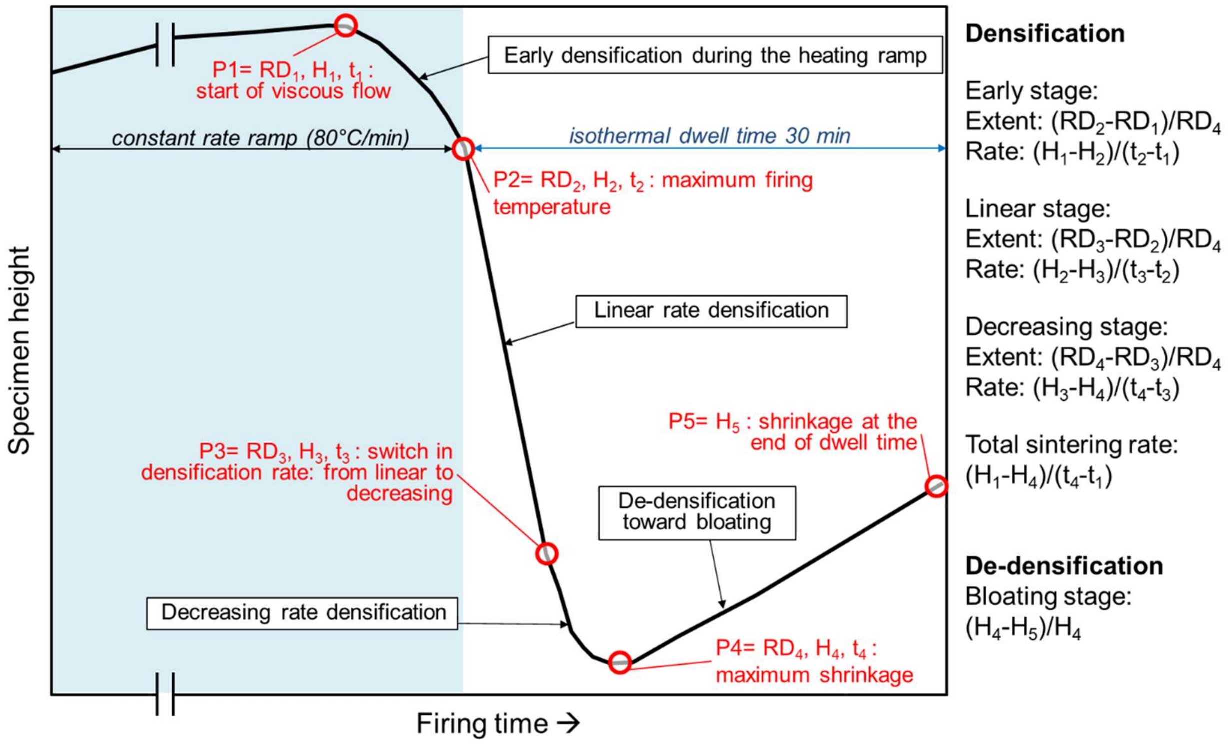

Figure S3. The curve describes the linear shrinkage as a function of firing time during the isothermal run. Different stages of sintering are pointed out, with the relative turning points. Since the mechanisms governing the viscous flow sintering are known, it is possible to describe the process through the parameterization of the firing curve. At temperatures around 1000 °C, a viscous mass transport begins with a given shrinkage (from P1 to P2). This early densification occurs during the heating ramp and is promoted by the flow of the neo-formed liquid phase. Once the sample reaches the maximum firing temperature, the sintering enters the isothermal stage (from P2 to P4), with a first linear rate (from P2 to P3) and then a decreasing rate (the final densification from P3 to P4). Once reached the maximum density, an inversion of the sintering process may take place, leading to a more or less accentuated expansion, with a bloating quantified from P4 to P5. (RD = relative density, H = height of the specimen and t = time at the point P*X*, with *X*: 1-5).

Table S2. Firing behavior of ceramic batches in the HSM tests.

	Firing T	Start of densification	Early stage extent	Linear stage extent	Decreasing stage extent	Early stage rate	Linear stage rate	Decreasing stage rate	Sintering rate (total)	Relative density at linear> decreasing switch	Efficiency of densification*	Relative density (max)	Bloating
**Unit**	**°C**	**°C**	**%**	**%**	**%**	**1/min**	**1/min**	**1/min**	**1/min**	**1**	**g/cm³**	**1**	**%**
**SOPO**	1100	1036	21%	9%	70%	0.53	0.14	0.02	0.04	0.713	1.948	0.754	0%
	1150	1054	10%	16%	73%	0.46	0.39	0.12	0.16	0.709	2.073	0.800	0%
	1200	1025	8%	23%	68%	0.49	1.30	0.20	0.28	0.718	2.291	0.876	0%
	1250	1054	12%	22%	65%	1.02	2.86	0.25	0.36	0.750	2.550	0.969	0%
**VICH**	1100	1039	14%	14%	72%	0.72	0.51	0.04	0.05	0.732	2.090	0.786	0%
	1150	1046	14%	19%	67%	0.54	0.47	0.11	0.16	0.746	2.226	0.840	0%
	1200	1054	12%	19%	68%	0.76	1.47	0.16	0.24	0.745	2.378	0.889	0%
	1250	1043	26%	22%	52%	1.08	3.26	0.18	0.33	0.791	2.503	0.926	0%
**POST**	1100	1085	7%	20%	74%	1.09	0.65	0.03	0.05	0.749	2.113	0.805	0%
	1150	1037	17%	20%	63%	0.78	0.72	0.11	0.18	0.762	2.263	0.859	0%
	1200	1040	25%	35%	41%	1.03	1.53	0.20	0.38	0.805	2.371	0.888	5.2%
	1250	1054	42%	30%	28%	1.26	3.59	0.38	0.80	0.837	2.381	0.894	8.4%
**GPOR**	1100	975	24%	18%	58%	0.62	0.49	0.06	0.10	0.700	1.940	0.745	0%
	1150	1008	14%	16%	71%	0.69	0.74	0.16	0.21	0.708	2.179	0.838	0%
	1200	1026	16%	20%	64%	0.77	1.98	0.35	0.49	0.726	2.383	0.913	3.3%
	1250	1014	32%	33%	36%	1.33	4.85	1.11	1.53	0.786	2.350	0.883	8.1%
**GSTO**	1050	958	26%	32%	42%	0.83	0.88	0.02	0.05	0.770	2.095	0.800	0%
	1100	952	43%	22%	35%	0.92	0.81	0.03	0.11	0.776	2.116	0.804	0%
	1150	938	55%	23%	21%	0.87	0.99	0.08	0.28	0.800	2.173	0.824	0%
	1200	980	54%	24%	21%	1.28	1.78	0.18	0.56	0.834	2.307	0.872	6.4%

*Efficiency of densification= (bulk density of unfired body/specimen volume at time t) × 100

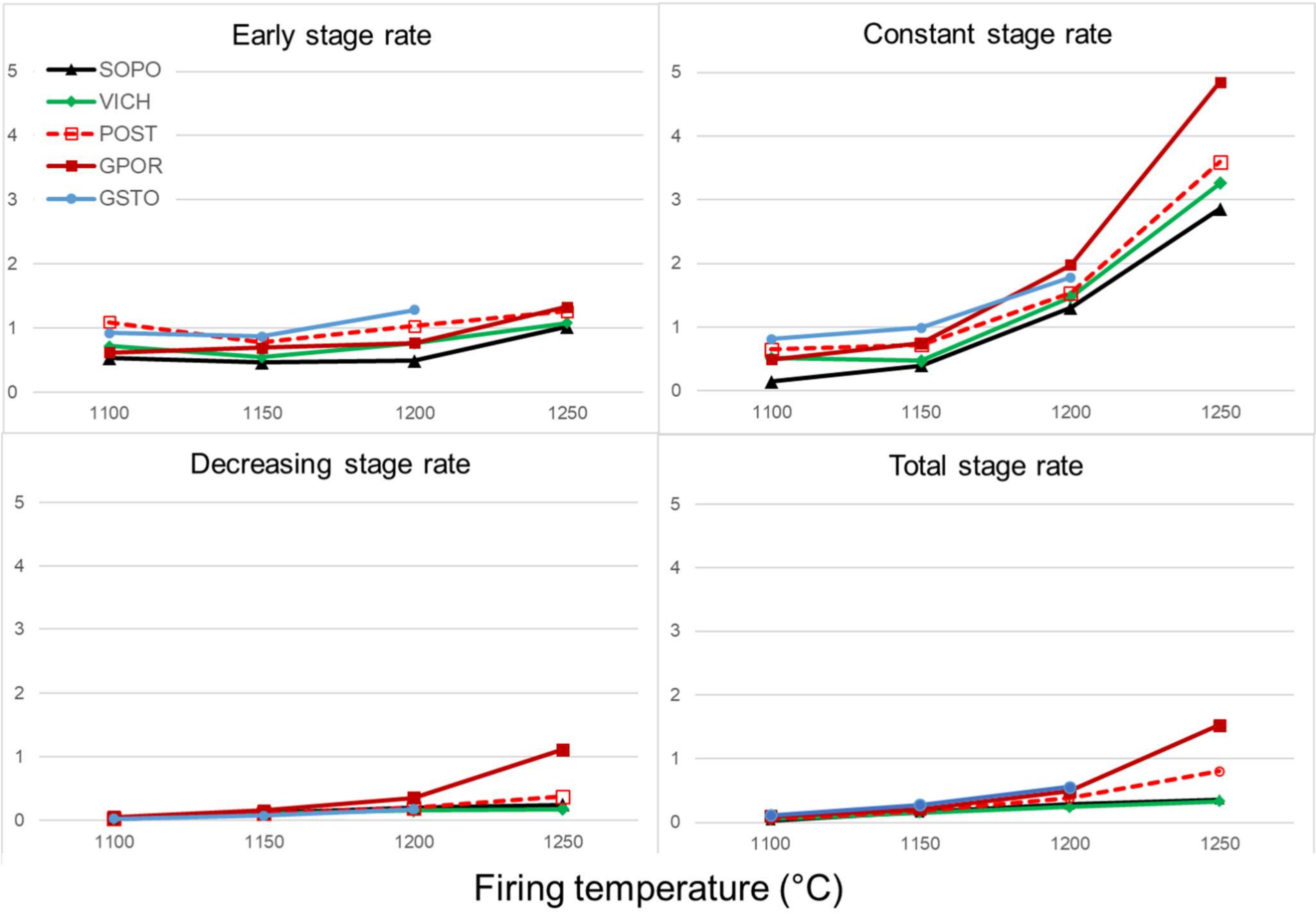

Figure S4. Sintering kinetics (min^{-1}) in the different stages of the process.

Table S3. Phase composition (% weight) of the bodies fired at different maximum temperatures.

Sample	T°C	Quartz	Mullite	Plagioclase	K-feldspar	Illite	Cristobalite	Amorphous
**SOPO**	**1000**	~27	~5	~22	~4	~6	-	~35
	**1100**	23.7	6.2	13.0	2.9	-	-	54.4
	**1180**	24.9	18.8	6.7	2.5	-	-	47.1
	**1200**	25.6	20.4	1.6	2.3	-	-	50.1
	**1250**	21.2	17.8	0.7	1.4	-	-	58.9
	**1280**	18.5	20.1	-	-	-	1.0	60.4
**VICH**	**1000**	~33	~4	~20	~3	~5	-	~36
	**1100**	29.3	1.5	11.1	2.4	-	-	55.8
	**1180**	28.5	6.8	7.4	-	-	-	57.4
	**1200**	26.2	17.9	2.1	1.5	-	-	52.4
	**1250**	24.2	12.8	0.6	0.0	-	-	62.4
	**1280**	18.6	15.6	-	-	-	1.3	64.5
**POST**	**1000**	~28	~3	~34	~4	~4	-	~27
	**1100**	24.4	2.6	26.4	4.0	-	-	42.6
	**1150**	22.5	3.4	19.3	1.3	-	-	53.5
	**1200**	23.0	3.5	6.3	1.2	-	-	66.0
	**1225**	20.4	3.0	3.5	1.9	-	-	71.2
	**1250**	18.4	4.0	0.5	2.9	-	-	74.1
**GPOR**	**1000**	~32	~2	~40	-	~6	-	~20
	**1100**	26.2	3.7	30.6	1.8	-	-	37.7
	**1150**	21.3	6.7	17.0	1.5	-	-	53.6
	**1200**	20.1	6.9	6.4	1.0	-	-	65.6
	**1250**	15.8	6.5	6.5	0.8	-	-	70.4
**GSTO**	**1000**	~24	~5	~28	~3	~3	-	~37
	**1100**	19.9	3.4	27.4	1.8	-	2.6	44.9
	**1150**	17.0	3.9	24.0	1.8	-	2.7	50.6
	**1200**	12.7	4.6	19.1	1.6	-	2.2	59.7

Experimental uncertainty ±0.2%

Table S4. Melt physical properties at high temperature.

	Firing T	Melt shear viscosity	Surface tension	Relative viscosity	Effective viscosity	Surface tension/shear viscosity ratio
**Unit**	**°C**	**Log_{10} Pa s**	**N/m**	**1**	**Log_{10} Pa s**	**μm/s**
**SOPO**	1000	4.06	0.44	36.77	5.62	38.61
	1100	5.19	0.39	8.33	6.11	2.54
	1180	5.21	0.35	13.69	6.35	2.17
	1200	5.03	0.34	11.03	6.08	3.18
	1250	4.71	0.34	6.32	5.51	6.56
	1280	4.58	0.33	16.97	5.81	8.55
**VICH**	1000	5.44	0.43	35.20	6.98	1.57
	1100	5.16	0.40	7.63	6.04	2.73
	1180	4.85	0.37	6.88	5.69	5.17
	1200	5.10	0.34	9.47	6.08	2.71
	1250	4.67	0.34	5.15	5.38	7.22
	1280	4.68	0.33	8.28	5.60	6.77
**POST**	1000	6.30	0.42	65.84	8.12	0.21
	1100	5.70	0.38	19.45	6.99	0.76
	1150	5.39	0.36	8.79	6.34	1.47
	1200	4.84	0.35	4.23	5.47	5.10
	1225	4.68	0.35	3.26	5.19	7.32
	1250	4.53	0.34	2.83	4.98	10.15
**GPOR**	1000	6.75	0.42	55.33	8.49	0.08
	1100	5.35	0.38	25.79	6.76	1.69
	1150	5.02	0.35	8.00	5.92	3.34
	1200	4.60	0.34	4.07	5.21	8.66
	1250	4.35	0.33	3.22	4.86	14.97
**GSTO**	1000	6.30	0.37	40.22	7.90	0.19
	1100	5.42	0.37	18.93	6.70	1.39
	1150	4.99	0.36	12.19	6.08	3.67
	1200	4.59	0.35	6.63	5.41	9.09
